# A Novel E3 Probiotics Formula Restored Gut Dysbiosis and Remodelled Gut Microbial Network and Microbiome Dysbiosis Index (MDI) in Southern Chinese Adult Psoriasis Patients

**DOI:** 10.3390/ijms24076571

**Published:** 2023-03-31

**Authors:** Chi Tung Choy, Un Kei Chan, Pui Ling Kella Siu, Junwei Zhou, Chi Ho Wong, Yuk Wai Lee, Ho Wang Chan, Joseph Chi Ching Tsui, Steven King Fan Loo, Stephen Kwok Wing Tsui

**Affiliations:** 1 Microbiome Research Centre, BioMed Laboratory Company Limited, Hong Kong, China; 2 Hong Kong Institute of Integrative Medicine, Faculty of Medicine, The Chinese University of Hong Kong, Hong Kong, China; 3 Dermatology Centre, CUHK Medical Centre, The Chinese University of Hong Kong, Hong Kong, China; 4 School of Biomedical Sciences, Faculty of Medicine, The Chinese University of Hong Kong, Hong Kong, China; 5 Centre for Microbial Genomics and Proteomics, The Chinese University of Hong Kong, Hong Kong, China; 6 Hong Kong Bioinformatics Centre, The Chinese University of Hong Kong, Hong Kong, China

**Keywords:** psoriasis, gut microbiome, metagenomics, Parabacteroides distasonis, gut dysbiosis index, probiotics

## Abstract

Psoriasis is a common chronic immune-mediated inflammatory skin disease with the association of various comorbidities. Despite the introduction of highly effective biologic therapies over the past few decades, the exact trigger for an immune reaction in psoriasis is unclear. With the majority of immune cells residing in the gut, the effect of gut microbiome dysbiosis goes beyond the gastrointestinal site and may exacerbate inflammation and regulate the immune system elsewhere, including but not limited to the skin via the gut-skin axis. In order to delineate the role of the gut microbiome in Southern Chinese psoriasis patients, we performed targeted 16S rRNA sequencing and comprehensive bioinformatic analysis to compare the gut microbiome profile of 58 psoriasis patients against 49 healthy local subjects presumably with similar lifestyles. *Blautia wexlerae* and *Parabacteroides distasonis* were found to be enriched in psoriasis patients and in some of the healthy subjects, respectively. Metabolic functional pathways were predicted to be differentially abundant, with a clear shift toward SCFA synthesis in healthy subjects. The alteration of the co-occurrence network was also evident in the psoriasis group. In addition, we also profiled the gut microbiome in 52 of the 58 recruited psoriasis patients after taking 8 weeks of an orally administrated novel E3 probiotics formula (with prebiotics, probiotics and postbiotics). The Dermatological Life Quality Index (*p* = 0.009) and Psoriasis Area and Severity Index (*p* < 0.001) were significantly improved after taking 8 weeks of probiotics with no adverse effect observed. We showed that probiotics could at least partly restore gut dysbiosis via the modulation of the gut microbiome. Here, we also report the potential application of a machine learning-derived gut dysbiosis index based on a quantitative PCR panel (AUC = 0.88) to monitor gut dysbiosis in psoriasis patients. To sum up, our study suggests the gut microbial landscape differed in psoriasis patients at the genera, species, functional and network levels. Additionally, the dysbiosis index could be a cost-effective and rapid tool to monitor probiotics use in psoriasis patients.

## 1. Introduction

Psoriasis is a common chronic T cell-mediated inflammatory skin disease with a worldwide prevalence of approximately 2–3% [1,2,3]. The southern Chinese population is reported to be less susceptible to psoriasis, with a local prevalence of 0.3–0.6% in Hong Kong [4,5]. According to the 2020 joint American Academy of Dermatology (AAD)—National Psoriasis Foundation (NPF) guidelines [6], psoriasis is not only a skin disease but also a chronic multisystem inflammatory disorder. About one-third of psoriasis patients develop psoriatic arthritis during their lifetime with stiffness, pain and swelling of joints, and it may progress to exhausting joint destruction with a dramatic deterioration in the quality of life [7,8,9,10]. On top of that, severe psoriasis patients with early onset are found to be at a higher risk for cardiometabolic comorbidities than the general population, including but not limited to vascular inflammation [11], coronary atherosclerotic plaques [12,13], type 2 diabetes [14,15], strokes [16], or myocardial infarction [17,18]. In addition, there has been consistent evidence that psoriasis is associated with gastrointestinal comorbidities, such as irritable bowel syndrome and inflammatory bowel disease [19,20].

Similar to other inflammatory diseases, the pathogenesis of psoriasis is complicated and has not yet been fully understood [6]. However, the overactivation of an adaptive immune system is believed to be central in psoriasis development [6,21], involving various cell types such as natural killer T cells, macrophages, myeloid dendritic cells, plasmacytoid dendritic cells, and keratinocytes. For example, activated T lymphocytes (Th1 and Th17) release pro-inflammatory interleukins (IL-1, IL-6, IL-17, IL-28, etc.), which trigger the inflammation cascade in psoriasis patients. Other than that, genetic factors are also major risk factors, such that the risk of psoriasis is approximately 40%, 14% and 6% if both parents or either parent or sibling are affected, respectively [22,23]. The histocompatibility complexes, HLA-Cw6 and HLA-Cw7, have been linked to erythematous, inflamed, and thickened skin in people with the disease [24,25,26]. On top of that, behavioral and environmental factors can also accelerate and trigger the onset of psoriasis [6], e.g., streptococcal infection, recent skin trauma, smoking and stress.

In addition, there have been growing amounts of evidence about the association between gut microbiota with psoriasis as an inflammatory disease, where gut microbial dysbiosis has been reported in patients with psoriasis and psoriatic arthritis [27,28,29,30,31,32,33]. This association was conceptualized and postulated as a “gut (-brain)-skin axis”, which provides valuable foundation and insights into the role of a symbiotic relationship between the gut microbiota and skin barrier via the modulation and maintenance of the host’s immune system [34]. It is also believed that gut microbes regulate immunological pathways via microbial-derived metabolites and products, which include, but not limited to short-chain fatty acids (SCFA), polysaccharides A and lipopolysaccharides (LPS) [35]. Microbes residing in the gut can also synthesize and modify the host secretion of neuroactive molecules, hormones, and neurotransmitters, e.g., serotonin, at varying degrees to crosstalk with the neuroendocrine system, which hampers the skin’s homeostasis. In general, gut dysbiosis with an altered abundance of *Akkermansia muciniphila*, *Staphylococcus aureus*, *Streptococcus pyogenes*, or *Candida albicans* was evidential and commonly reported in psoriasis patients [27,30,31]. Nonetheless, it is commonly known that gut flora composition varies with numerous environmental factors, such as geographical location, diet, and lifestyle. Hence, the changes in the microbiome profile in psoriasis patients may not be similar across different populations.

With the recognition of the role of dysbiosis in psoriasis, probiotics have been explored as a potential approach to restore microflora balance in an effort to alleviate cutaneous symptoms in psoriasis patients [36,37]. A handful of clinical trials have explored the efficacy of probiotics (*Bifidobacterium infantis*, *Bifidobacterium longum*, *Bifidobacterium lactis*, *Enterococcus*, *Lactobacillus rhamnosus*) as the treatment of psoriasis with a modest improvement in the Psoriasis Area and Severity Index (PASI) [38,39,40,41]. Although a recent meta-analysis concluded that probiotics and the control group did not yield statistical significance (*p* = 0.11) [42], possibly due to increased compliance in both groups after enrolment into clinical trials [37,43], probiotics demonstrated potentially positive effect with minimal adverse effects in psoriasis patients and warranted further studies.

In this study, our group aimed at exploring alterations in the gut microbiome profile between psoriasis patients and healthy subjects in Southern Chinese people with closer lifestyles by targeted 16S rRNA sequencing. We then evaluate the effectiveness and gut microbiome evolution upon the application of a novel E3 synbiotics mixture of prebiotics, probiotics and postbiotics. These findings could help to evaluate, refine, and improve the clinical efficacy of probiotics as an intervention in psoriasis patients.

## 2. Results

### 2.1. Study Cohort

A total of 58 subjects aged between 18 and 65 years old with chronic plaque psoriasis and 49 healthy subjects without inflammatory skin diseases were prospectively recruited into the cohort through the Hong Kong Psoriasis Patients Association. The diagnosis and severity of psoriasis were further evaluated by a board-certified dermatologist (S.K.F.L.). The demographic and disease characteristics are summarized in Table 1. In brief, there was no statistically significant difference in age distribution between psoriasis and control group (mean age—psoriasis: 44.4 years, control: 46.3 years; *p* = 0.4413). The mean weight (psoriasis: 71.8 kg, control: 64.3 kg; *p* = 0.0226) and BMI (psoriasis: 25.8 kg/m^2^, control: 23.7 kg/m^2^; *p* = 0.0278) were slightly higher in the psoriasis group (which agreed with previously reported observation studies [44,45,46]), there was also a statistically significant discrepancy in gender between the two groups, but this did not significantly impact the gut microbiome composition as reflected by the adonis test (Table S1). Therefore, based on the adonis test result, we did not control for the weight or gender discrepancy during subsequent analysis.

### 2.2. Significant Difference in Gut Microbiome Composition between Psoriasis and Control Group

**Figure 1:**
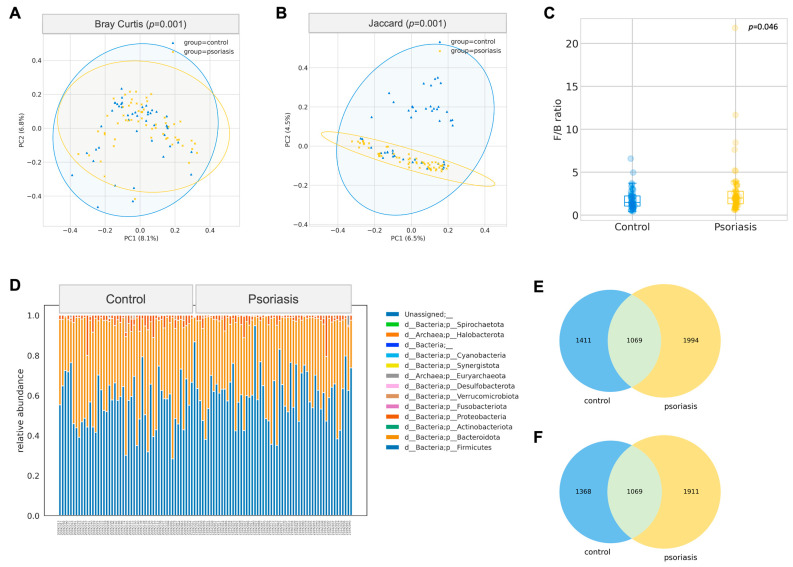
Gut composition profile of psoriasis patients. (**A**,**B**) Principal Coordinate Analysis biplot based on Bray–Curtis and Jaccard distances (PERMANOVA, 999 permutations). (**C**) Boxplot of Firmicutes/Bacteroidetes (F/B) ratio (Mann–Whitney U test). (**D**) Relative abundance of phyla. (**E**) Venn diagram of all ASV between control and psoriasis group. (**F**) Venn diagram of ASV (excluding rare ASV) between control and psoriasis group.

The gut microbiome composition of participants with psoriasis was significantly different from the apparently normal group in terms of Bray–Curtis (*p* = 0.001, PERMANOVA) and Jaccard distance (*p* = 0.001, PERMANOVA), which was demonstrated by the principal coordinates analysis biplot (Figure 1A,B). This difference was not due to a change in alpha diversity, including richness, Chao1 index, Faith’s phylogenetic diversity, Shannon, or Inverse Simpson, as shown in Figures S1 and S2.

**Figure 2:**
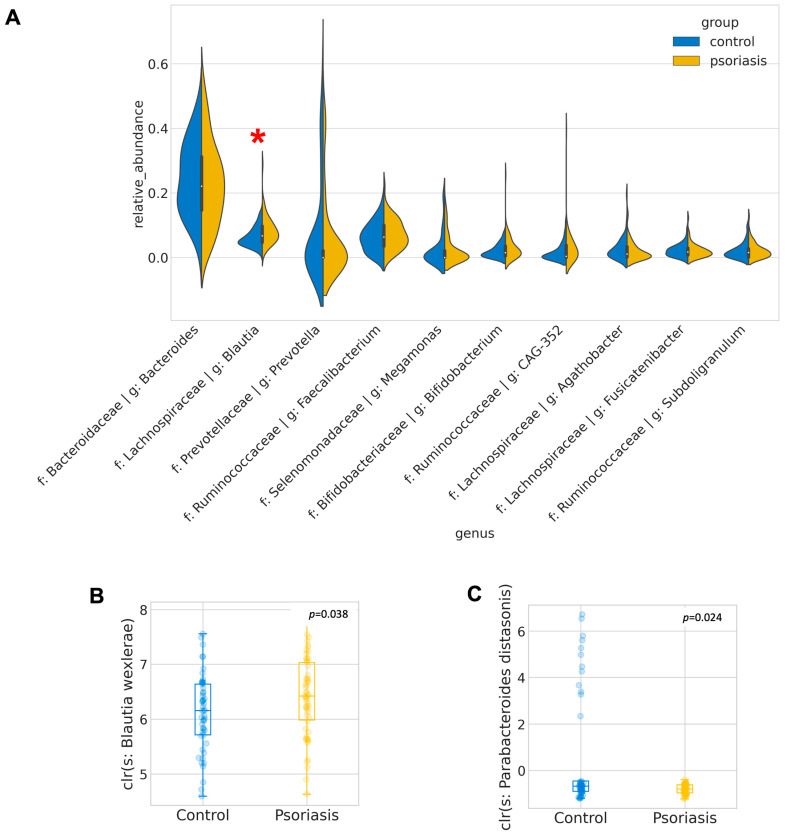
Most abundant and differentially abundant genera. (**A**) Distribution of relative abundance of the 10 most abundant genera. Differentially abundant genera are indicated by red asterisk. (**B**,**C**) Boxplot of center-log-ratio (clr) transformed abundance of *Blautia wexlerae* and *Parabacteroides distasonis*.

A total of 4474 unique amplicon sequence variants (ASV) were identified, of which 126 ASVs were categorized as rare ASVs with exactly one count through the dataset. After alignment, these ASVs were assigned to 12 phyla, 269 genera and 622 species. The Firmicutes/Bacteroidetes ratio was marginally increased in the psoriasis group (*p* = 0.046, Mann–Whitney U), but no other significant difference at the phylum level was identified. The most abundant genera were *Bacteroides*, *Blautia*, *Prevotella*, *Facalibacterium*, *Megamonas*, *Bifidobacterium*, *Ruminococcaceae CAG-352*, *Agathobacter*, *Fusicatenibacter*, *Subdoligranulum*. Among these genera, *Blautia* was found to be significantly enriched in psoriasis patients (*p* = 0.015, Mann–Whitney-U). ANCOM was performed to discover a differential abundance at the ASV level. A total of 25 ASVs, which were assigned to seven named species and eight unnamed species from 11 genera, were found to be differentially expressed among the two groups, as detailed in Table 2. Among the differentially abundant ASVs, the signal of *Blautia wexlerae* was further augmented with the Mann–Whitney U test (*p* = 0.038) on its relative abundance. With *B. wexlerae* being the most abundant species within *Blautia*, it is highly likely that *B. wexlerae* is the main contributor to the increase in *Blautia* observed at the genus level. More subjects from the control group harbored a higher percentage of *Parabacteroides distasonis* (center-log ratio transformed) (*p* = 0.024, Mann–Whitney-U, Figure 2, Table S2).

### 2.3. Effect of 8-Week Probiotics Intake in the Gut Microbiome Composition in Psoriasis Group

**Figure 3:**
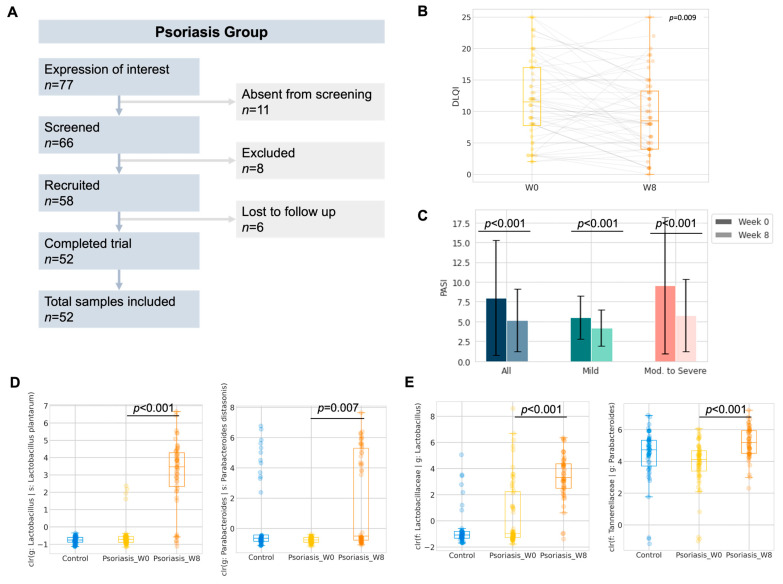
Pilot study of 8-week probiotics in psoriasis group (**A**) Study flow diagram. (**B**) Boxplot of the Dermatology Life Quality Index (DLQI) of the participants. (**C**) Change in Psoriasis Area and Severity Index (PASI) of the participants. (**D**,**E**) Boxplot of center-log-ratio (clr) transformed abundance of *Lactobacillus plantarum* and *Parabacteroides distasonis*; *Lactobacillus* and *Parabacteroides* (Wilcoxon signed rank).

Six subjects were lost to follow-up after recruitment, and a total of fifty-two subjects with psoriasis were included in further analysis (Figure 3A). No adverse effect was reported nor recorded throughout the study period. No substantial weight gain or loss which warranted recording was described by the participants during the study period. The Dermatological Life Quality Index (DLQI) (∆DLQI = −2.3 ± 6.3, *p* = 0.009, Figure 3B) and Psoriasis Area and Severity Index (PASI) (∆PASI = −2.9 ± 5.1, ∆%PASI = −24.5 ± 26.3%, *p* < 0.001, Figure 3C) were significantly improved after taking 8 weeks of oral administration of probiotics. The details of PASI or DLQI improvement regarding disease severity are included in Table S7. We further stratified the patients into subgroups by disease severity and responsiveness, where patients who were either currently receiving systemic therapies or PASI > 10 at the baseline were regarded as having moderate to severe psoriasis. Subjects who showed a significant improvement in PASI or DLQI (e.g., ∆DLQI < −3.3) were regarded as responders towards 8-week probiotics treatment. The responsiveness and objective improvement in BSA, DLQI or PASI were found to be independent of disease severity (Table 3). Therefore, subsequent analysis of the gut microbiome was performed without further stratification.

While the objective improvement in DLQI or PASI could have been introduced by the E3 probiotics formula through the restoration of gut dysbiosis, we compared the gut microbiome profile of the participants after 8-weeks of probiotics intake with respect to their baseline profile (Figures S3 and S4). To interrogate the impact of probiotics on the gut microbiome, ANCOM at the ASV level was performed as the exploratory analysis of differentially abundant units. Five differentially abundant ASVs, which belonged to three named species and two unnamed species from five genera, were identified (Table 4). Two out of five ANCOM identified differentially abundant species, namely *Lactobacillus plantarum* (adjusted *p* < 0.001, Wilcoxon signed rank, Benjamini-Hochberg correction) and *Parabacteroides distasonis* (adjusted *p* = 0.007, Wilcoxon signed rank, Benjamini-Hochberg correction), which were further confirmed by the Wilcoxon signed rank test on the center-log ratio transformed abundance from the paired samples. The increase in *Lactobacillus plantarum* abundance could likely have contributed to the intake of a probiotics mixture rich in *Lactobacillus*.

In addition, we also performed the taxonomic analysis at the phylum and genus level by the Wilcoxon signed rank test with Benjamini–Hochberg correction on the center-log ratio transformed abundance from paired samples. Phylum Synergistota (adjusted *p* = 0.008, Wilcoxon signed rank, Benjamini–Hochberg correction) was found to be significantly decreased (Figure S5). On the other hand, *Lactobacillus* (adjusted *p* < 0.001, Wilcoxon signed rank, Benjamini–Hochberg correction) and *Parabacteroides* (adjusted *p* < 0.001, Wilcoxon signed rank, Benjamini–Hochberg correction) were significantly increased after 8 weeks of probiotics intake (Figure 3 and Figure S6).

### 2.4. Depleted SCFA Related Functional Abundance in Psoriasis Group

**Figure 4:**
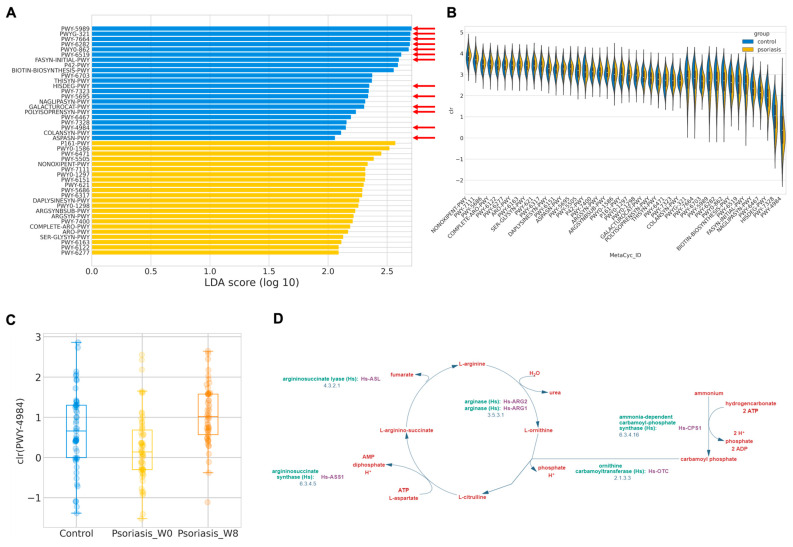
Predicted MetaCyc pathways abundance. (**A**) Log LDA score of differentially abundant MetaCyc pathways. Pathways related to short chain fatty acids (SCFAs) synthesis is indicated by a red arrow. (**B**) Distribution of center-log-ratio (clr) transformed abundance of the differentially abundant MetaCyc pathways. (**C**) Boxplot of center-log-ratio (clr) transformed abundance of PWY-4984. (**D**) Pathway diagram of PWY-4984 from MetaCyc.

In order to investigate the functional abundance, which was postulated to more accurately reflect the physiological consequence of gut microbiome profile, functional abundance was in silico inferred by PICRUSt2 following LefSe. A total of 44 discriminative features were identified with an absolute LDA larger than two (Figure 4A,B). The majority of the features over-represented in the control group were related to short-chain fatty acid (SCFA) synthesis or metabolism, such as stearate, oleate, palmitoleate, mycolate, (5Z)-dodecenoate, L-histidine. By contrast, 22 MetaCyc functional pathways involved in L-arginine biosynthesis were exacerbated in psoriasis patients, as detailed in Table 5.

Similar procedures have been employed in the prediction of the functional abundance in the psoriasis group, and after taking 8 weeks of probiotics, there are only two discriminative features found—PWY-2326 (GDP-mannose biosynthesis) enriched at the baseline (log LDA = 2.106, *p* = 0.0357) and PWY-4984 (urea cycle, Figure 4D) enriched at week 8 (log LDA = 2.352, *p* < 0.001). Interestingly, PWY-4984 was also found to be enriched in the control group. The predicted abundance of this pathway is summarized in Figure 4C. It may hint at the potential functional mechanism of how probiotics alleviate disease severity in psoriasis patients.

### 2.5. Remodelling of Microbial Co-Occurrence Network

**Figure 5:**
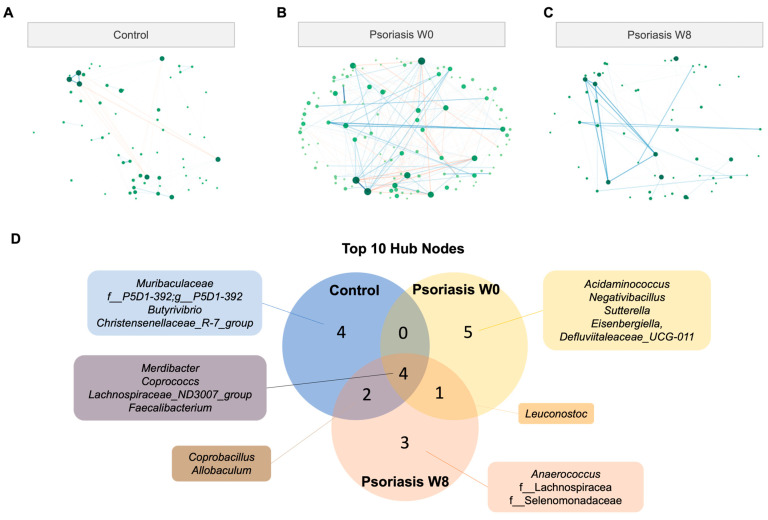

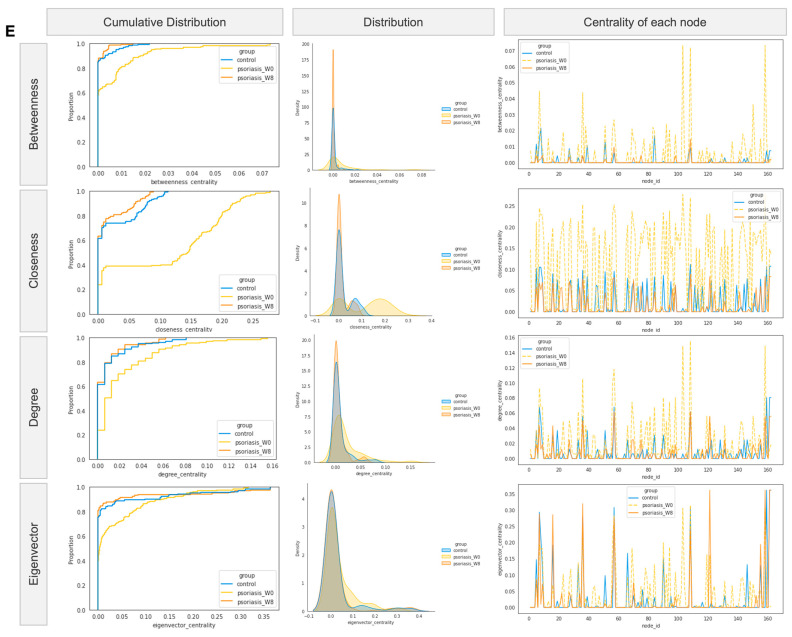
Co-occurrence/co-exclusion network. (**A**,**B**,**C**) Co-occurrence network diagram of control and psoriasis group. (**D**) Venn diagram of top 10 hub nodes across networks. (**E**) Empirical cumulative distribution function (eCDF) (**left**) and kernel density distribution (**middle**) of betweenness, closeness, degree, and eigenvector centrality measures and centrality of each node (**right**).

Compared with the co-occurrence/exclusion network in the control group, the gut microbial network was clearly remodeled (Figure 5, Tables S3–S5). For an easier comparison and better visualization, the topology of the nodes was fixed, such that the lower left node in Figure 5A could represent the same taxonomic unit as the lower left node in Figure 5B. Each node represents a taxonomical unit at the genus level, while each connection (i.e., edge) represents a significant positive (co-occurrence) or negative (co-exclusion) association in terms of abundance between the connecting nodes as determined by SPIEC-EASI. The node size is proportional to the respective number of degrees, while the edge width is proportional to the strength of association. A blue and red labeled edge represents a positive and negative association, respectively.

There were 162 nodes in total, and the total number of edges was 104 and 281 in the psoriasis and control groups, respectively. The number of edges in the psoriasis group in week 8 was reduced to 85. The connection between the nodes was obviously disrupted in the psoriasis group. The top 10 hub nodes (the node with the highest degree of connections) accounted for 96.2% (100/104), 63.3% (178/281), 96.5% (82/85) of the connections in the network of the control group, psoriasis baseline and psoriasis week 8, respectively. Therefore, the psoriasis baseline network was more dispersive and distributed in nature. Only four out of the top ten hub nodes were shared across all groups, namely *Merdibacter*, *Coprococcs*, *Lachnospiraceae_ND3007_group* and *Faecalibacterium* (Figure 5D). Apart from the above-mentioned central hub nodes, *Coprobacillus* and *Allobaculum* were shared between the control and psoriasis at week 8 only, while *Leuconostoc* was shared by the psoriasis group across time points. There were more shared hub nodes between the psoriasis week 8 network with the control group (6/10) than the psoriasis baseline network (4/10).

The difference in networks was further supported by centrality measures (including betweenness, closeness and eigenvector) other than the degree measure, as shown in Figure 5E. The network centralities measured the psoriasis group at week 8 (colored in orange) resembled that of the control group (colored in blue) more than its baseline (colored in yellow) considering the cumulative distribution of measure (left), the density distribution of measure (middle) and centrality of each node (right).

### 2.6. Development of Psoriasis Specific Machine Learning Based Microbiome Dysbiosis Index (MDI)

Following the above promising results of gut dysbiosis in psoriasis, our group aimed at developing a lifestyle-neutral gut dysbiosis test in differentiating the psoriasis and control group. To avoid social and geographical bias in the gut microbiome, 10 microbial markers were selected as targets based on previously reported microbial species enriched in non-healthy subjects from a stool metagenomics analysis of 4347 individuals [47]. Quantitative PCR (qPCR) was performed on the remaining samples in the dataset, including 41 out of 49 data points from the control group, 56 out of 58 baseline data points from the psoriasis group, and 39 out of 52 post-8-week probiotics intake data points from the psoriasis group.

To construct a dysbiosis index from the qPCR result, a machine learning approach was utilized, as detailed in the above methodology Section 4.6. In brief, 25% of the data were held out as the testing set, while 4-fold cross-validation on the training set was adopted to estimate the performance in terms of F score (a combination of precision and recall) and the area under the receiver operating characteristic curve (AUROC; combination of sensitivity and specificity) across five machine learning algorithms, namely logistics regression (LR), support vector machine (SVM), random forest (RF), extreme gradient boosting (XGB), and light gradient boosting machine (LGBM). RF and LGBM were the top-performing models (F score = 0.89, AUROC = 0.88) (Figures S8–S10), and RF was chosen for subsequent analysis due to its wide adoption in microbiome research [48].

**Figure 6:**
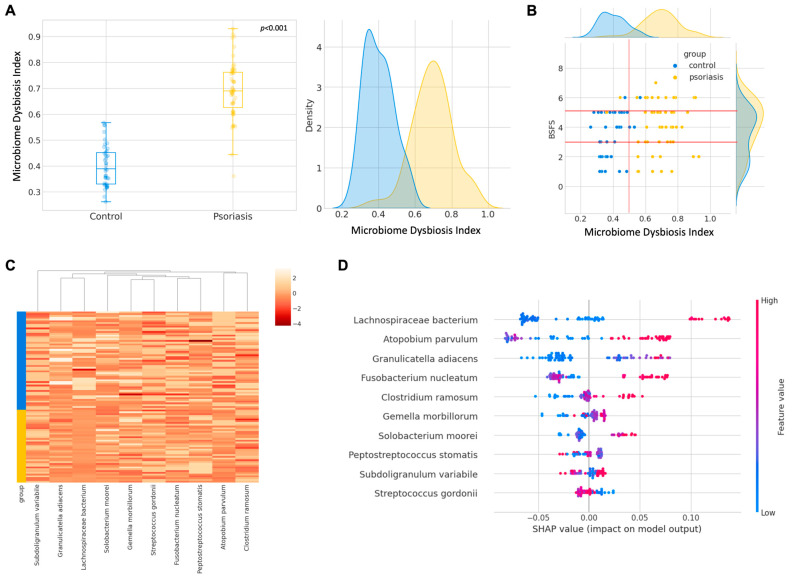
Development of machine learning derived gut microbiome dysbiosis index (MDI) (**A**) Boxplot and kernel density distribution of the gut dysbiosis index. (**B**) Biplot of MDI with Bristol Stool Form Scale (BSFS). (**C**) Heatmap of log 2 ∆Ct stratified by group. (**D**) Feature importance of each microbial marker on random forest model output.

The machine learning-based dysbiosis index (MDI) [range: 0–1] could clearly distinguish the control and psoriasis group with a cut-off of 0.5 and with the distribution of the index summarized in Figure 6A. Interestingly, the dysbiosis index was seemingly weakly and positively correlated with the Bristol Stool Form Scale (BSFS) within each group (Figure 6B), but more data are required to draw a definitive correlation. The log2∆Ct values stratified by the group are shown in Figure 6C, and the plain expression without the aid of dysbiosis index was not straightforward in classifying psoriasis subjects from the control group. We also evaluated the feature importance in terms of the SHAP value, as reflected in Figure 6D. Among all the targets, *Lachnospiraceae* bacterium, *Atopobium parvulum*, *Granulicatella adiacens*, and *Fusobacterium nucleatum* were found to impose a higher impact on the dysbiosis index calculation. Except for *Granulicatella adiacens*, the abundance of *Lachnospiraceae* bacterium, *Atopobium parvulum*, and *Fusobacterium nucleatum* were also consistently influential in the index calculation if employing LGBM and XGB algorithms (Figure S9). With the insights into important features, we tried to fit the machine learning models with the fifth most important features. Reducing the dimensionality of data seemingly improved their performance during training, but the improvement did not translate into a testing procedure. Even though the performance of LGBM trained with a smaller dataset was similar, there was a slight drop in both the F score and AUROC for RF (Figure S8). Therefore, RF trained with all the features was deployed.

### 2.7. Microbiome Dysbiosis Index (MDI) Correlates with PASI Responsiveness

**Figure 7:**
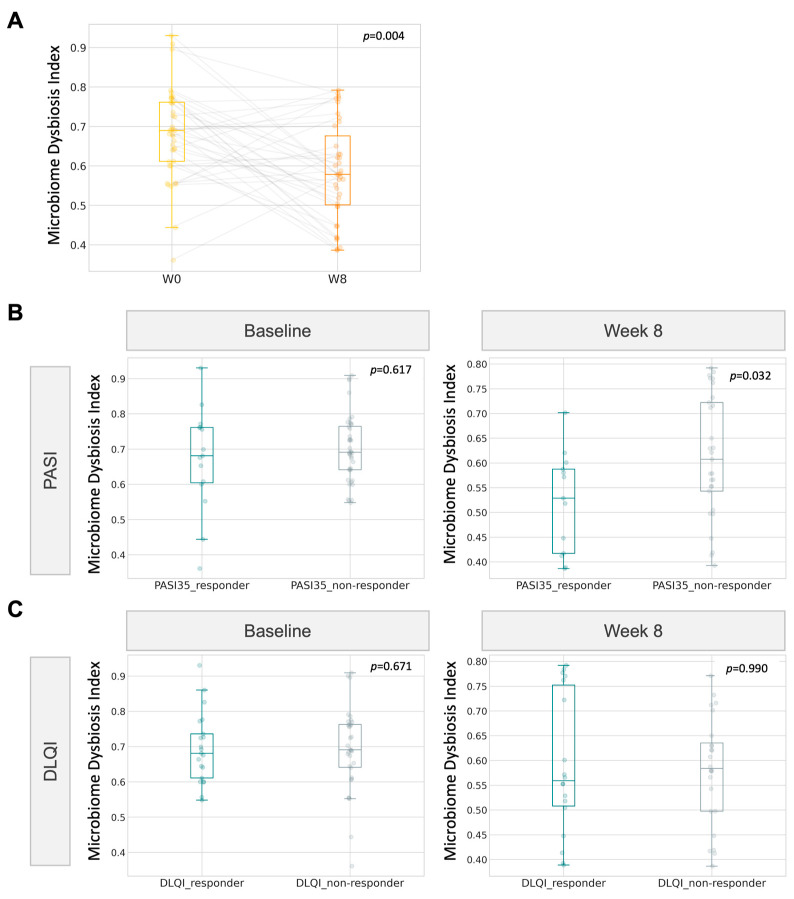
Correlation of gut microbiome dysbiosis index (MDI) with clinical improvement (**A**) Boxplot of MDI for psoriatic participants at baseline and at week 8. (**B**,**C**) MDI of responders towards 8-week probiotics treatment.

Based on the improvement in the psoriasis group in reference to DLQI and PASI, we examined whether the microbiome dysbiosis index (MDI) could be exploited as a tool to monitor the progress of gut dysbiosis when receiving microbiome probiotics therapy. In line with the postulated lesser extent of gut dysbiosis in the psoriasis group after 8 weeks of probiotics intake, the gut dysbiosis index also followed a similar significant decline. At week 8, the index was found to be significantly lower (*p* = 0.032, Wilcoxon signed rank, Figure 7) in PASI responders than in non-responders.

## 3. Discussion

Despite the use of effective biologics therapies in the management of psoriasis over the past few decades, the exact triggers for underlying immunological events are still unclear [49]. The linkage between the gut and skin is gaining more and more attention as gut microbiome dysbiosis may play an important role in the pathogenesis of various autoimmune diseases. Our study aims to clarify the discrepancy in the gut microbiome profile at the taxonomic, functional and network levels. To the best of our knowledge, this is the first gut microbiome study comprising psoriasis patients in Hong Kong. The impact of diet on the gut microbiome composition is commonly recognized [50,51,52,53]. Therefore, it is believed that the incorporation of gut microbiome data across different geographical locations and lifestyles could expand and benefit the understanding of the complex interplay between gut flora and host health in general.

We reported a significant differential gut microbiome profile between psoriasis patients and the control group. This difference could be briefly explained at the phylum level by the Firmicutes/Bacteroidetes ratio and augmented by the differential abundance analysis at the genera level. Of note, *Blautia* genera (*B. wexlerae*), a genus composed of obligate anaerobic intestinal commensal microorganisms belonging to the *Lachnospiraceae* family [54,55], was significantly enriched in the gut of psoriasis patients. Similar findings were reported by another group [56]. However, it is uncertain if the difference observed in *B. wexlerae* was a consequence of other factors (e.g., weight, BMI) or due to the presence or development of psoriasis. The exact role of the *Blautia* genus in psoriasis remains unknown and controversial with contradictory associations reported in liver cirrhosis, colorectal cancer, and early-stage breast cancer [57,58,59]. On the other hand, *Parabacteroides distasonis*, which increased occurrence in the control, was another potential key player of psoriasis. This observation was also recapitulated by other groups previously in the mice model and a population other than southern Chinese [31,60,61,62]. It has been reported to possess anti-inflammatory properties, with emerging in vitro and in vivo evidence to alleviate colonic inflammation [63,64,65,66]. Most importantly, the abundance of *P. distasonis* improved after the 8-week oral administration of the novel E3 probiotics formula, reinforcing its key role in psoriasis. A recent study by Zhou’s group on collagen-induced arthritic (CIA) mice provided convincing evidence that oral feeding live *P. distasonis* could restrain Th17 cell differentiation [67], where Th17 is also an important mediator of psoriasis pathogenesis. Zhou’s group further recapitulated these effects by treating CIA mice with live *P. distasonis* derived from lithocholic acid (LCA), deoxycholic acid (DCA), isolithocholic acid (isoLCA) and 3-oxolithocholic acid (3-oxoLCA). COLANSYN-PWY (colanic acid building blocks biosynthesis), which was also found to be enriched in the control group inferred by PICRUSt2 and LefSe (Figure S7).

Furthermore, subsequent analysis revealed the potential explanation of the difference in the gut microbiome profile at the functional pathway and network level. Most of the discriminative features enriched in the control group were identified by linear discriminant analysis and related to the short-chain fatty acids (SCFAs) biosynthesis/degradation pathway. SCFAs—butyrate, acetate, propionate—are well known for their ability to inhibit both adaptive and innate immune responses via reducing the proliferation, migration, and pro-inflammatory cytokine production of various immune cells, such as Th17, Treg, DCs [68,69,70,71]. It is an important class of metabolites that mediate the distal effects of gut microbiota in the host health status [72]. Some researchers unveiled that SCFAs could maintain gut barrier integrity and reduce intestinal permeability so that microbial dysbiosis related to the deregulation of SCFA may associate with “leaky gut” [70,71], and thus lead to the onset of numerous diseases including but not limited to psoriasis. Furthermore, a number of studies showed that an altered intestinal barrier was linked with psoriasis activity and severity through quantifying intestinal barrier integrity markers (such as claudin-3 and intestinal fatty acid binding protein, I-FABP) or the serum concentrations of gut microbiota-associated metabolite trimethylamine N-oxide (TMAO) [73,74,75,76,77]. The enrichment of L-arginine-related pathways and urea cycle intermediates (e.g., citrulline, ornithine, proline) also coincided with previous serum metabolomic studies on psoriasis patients [78]. The extent of this contribution in regulating blood metabolites by the gut microbiome in psoriasis has yet to be determined, but it certainly implies a plausible mechanism for the effect of the gut microbiome in people with psoriasis.

In addition to in silico pathway analysis, the co-occurrence/-exclusion network presented a paramount piece of evidence for microbial dysbiosis in the gut of psoriasis patients. It is obvious that connection patterns in the network were remodeled with a clear manifestation in centrality measures. However, the driving force behind this remodeling remains unknown, and it may be due to the relative abundance changes in individual species or the disturbed balance of the whole gut flora. Additionally, the interpretation of the network analysis result is not always direct and straightforward; no gold standard comparison methodology has existed yet [79,80,81]. Nonetheless, it does not undermine the observed distinction of a co-occurrence network between psoriasis and the apparent normal group and partial restoration of gut dysbiosis in psoriasis after taking 8 weeks of oral probiotics [79].

Leveraging the observed gut dysbiosis in this cohort, we further explored the opportunity to monitor the level of gut dysbiosis by leveraging cost-effective qPCR and machine learning techniques. The machine learning-derived gut dysbiosis index demonstrated excellent precision and recall relying on 10 carefully selected microbial markers based on big data to minimize lifestyle bias in a targeted discovery. Despite the correlation between the index and disease severity, this could be affected by a number of confounding factors, and the index was shown to reflect the treatment outcome of the microbiome probiotics therapy in psoriasis patients with a favorable PASI response. It warrants further investigation of the index as a practical and objective tool in the monitoring of gut dysbiosis status while taking probiotics.

Taken together, our findings provide evidence of gut microbiome remodeling in southern Chinese adult psoriasis patients at the baseline and after a course of orally administrated probiotics with the taxonomical to pathway and network level. Even though it is expected that these findings could be discordant from previous studies with psoriasis from different demographic backgrounds, *B. wexlerae* was also found to contribute to psoriasis pathology in our cohort. A detailed mechanism of how *B. wexlerae* regulates immune response is largely unresolved [54] and requires further investigations into the underlying principle to reveal new insights into the development and progression of various immune diseases. *P. distasonis* is another crucial player in alleviating psoriasis severity by gut microbiome modulation. Comprehensive bioinformatics analysis highlighted the connection between gut microbiota dysbiosis and psoriasis, with SCFAs as a probable mediator. Of note, our current study was limited by the short 8-week study period. Further investigation with a longer follow-up period would be warranted to explore the gut microbiome evolution, stability and resilience after longer-term administration and the discontinuation of probiotics therapy. Nonetheless, with the recent development of a microbiome-targeted therapeutics approach in managing various disorders [82,83,84,85,86,87,88,89], our study hints at the possibility and provides a scientific basis to leverage the microbiome as part of the management of psoriasis in patients.

## 4. Materials and Methods

### 4.1. Subject Recruitment and Study Design

A total of 58 adults (18–65 years old) with psoriasis and 49 adult subjects without known dermatological disorders of Chinese ethnicity were recruited through the collaboration between the Hong Kong Psoriasis Patient Association, The Chinese University of Hong Kong and BioMed Microbiome Research Centre. All participants (1) aged above 18 and (2) who provided informed consent were included. All psoriasis patients with any one of the following conditions were not recruited or were excluded from the study: (1) a history of adverse reaction to probiotics; (2) known overt bacterial infections in the skin; (3) known pregnancy; (4) premorbid medical conditions, such as cardiovascular, liver or renal dysfunction or diabetes mellitus; (5) having used oral corticosteroids, oral antibiotics, other immunosuppressive or any preparation of oral herbal medicines for the treatment of psoriasis in the past month; (6) having been diagnosed with atopic dermatitis, scabies, allergic contact dermatitis or seborrheic dermatitis; and (7) having taken anti-coagulant or anti-platelet drugs in the past month. All patients involved in this study were first diagnosed with psoriasis and evaluated by a board-certified dermatologist according to the Psoriasis Area and Severity Index (PASI) and Dermatological Life Quality Index (DLQI). Fecal samples were collected for downstream sequencing. All subjects were allowed to continue their usual medication or topical maintenance therapy for psoriasis during the trial. This study was conducted according to the guidelines of the Declaration of Helsinki and was approved by the Research Ethics Committee of the Hong Kong Doctors Union (protocol number HKSGM-2020AD-Study-protocol-vl-20220211).

### 4.2. Library Preparation and 16S rRNA Sequencing

All the fecal samples were homogenized in PurSafe^®^ DNA and RNA preservative (Puritan, Pittsfield, ME, USA) and were subjected to beating with 425–600 μm glass beads (Sigma-Aldrich, Saint Louis, MO, USA) for 1 h following the manufacturer’s instructions. A DNeasy Blood & Tissue Kit (Qiagen, Hilden, Germany) was used to conduct the isolation of microbial DNA from the fecal samples. The extracted DNA concentration of each sample was quantified using a Qubit™ dsDNA HS Assay Kit (Life Technologies, Carlsbad, CA, USA) with Qubit 3 Fluorometer (Thermo Fisher Scientific, Waltham, MA, USA). An amplicon library was constructed with 515F (5′-GTGCCAGCMGCCGCGG-3′)/907R (5′-CCGTCAATTTCMTTTRAGTTT-3′) and primer pair spanning targeting at aV4–V5 hypervariable of 16S rRNA genes, together with adapter sequences, multiplex identifier tags, and library keys. The 16S rRNA gene sequencing was performed using the Illumina MiSeq platform (Illumina, Inc., San Diego, CA, USA) following the original Earth Microbiome Project Protocols [90]. Index barcodes and adapters sequences were removed from pair-ended demultiplexed reads for downstream analysis.

### 4.3. Probiotic Mixture

All AD patients received a daily capsule of a novel E3 probiotics formula developed by BioMed Microbiome Research Centre (BioMed Laboratory Company Limited, Hong Kong) containing a mixture of 8 types of highly effective gastro-resistant probiotics (not less than 2 × 10^11^ CFU/capsule at the time of production), effective postbiotic HK-LP (heat-killed *Lactobacillus plantarum*, 10 mg/capsule), and triple prebiotics containing inulin (22 mg/capsule), Galacto-oligosaccharides (GOS) (8.1 mg/capsule), and Fructo-oligosaccharides (FOS) (0.9 mg/capsule) for eight weeks. The probiotics mixture was composed of *Lactobacillus acidophilus* GKA7, *Lactobacillus brevis* GKL9, *Lactobacillus casei* GKC1, *Lactobacillus gasseri* GKG1, *Lactobacillus reuteri* GKR1, *Lactobacillus plantarum* GKM3, *Bifidobacterium bifidum* GKB2 and *Bifidobacterium longum* GKL7. The postbiotics HK-LP involved in this formula was proved to enhance the probiotics functions. Moreover, prebiotics acts as an energy source for probiotics, which not only enhance the probiotic’s function but also foster intestinal peristalsis as well as detoxification.

### 4.4. Bioinformatics Analysis

Microbiome bioinformatics data were analyzed using a plugin-based system, QIIME 2–2022.2 [91], integrating various microbiome analysis algorithms and tools. Demultiplexed reads were quality controlled and denoised with DADA2 [92] using the q2-dada2 plugin to retrieve exact amplicon sequence variants (ASVs) [93]. All ASVs were then aligned by mafft [94], and then a phylogenetic tree was generated using fastree2 [95] via the q2-phylogeny plugin. The taxonomic annotation of the resulting ASV was carried out using the q2-feature-classifier plugin [96] and a pre-trained Naive Bayes classifier which was based on SILVA v138 taxonomic reference database with 99% similarity [97,98]. We used six metrics to indicate alpha diversity: the Observed OTUs, Chao1 Index (Chao1), ACE Index (ACE), Shannon Diversity Index (Shannon), Simpson Index (Simpson), and Faith’s phylogenetic diversity (PD). In addition, beta diversity was calculated based on the Jaccard distance metric, Bray–Curtis distance metric, weighted UniFrac, and unweighted UniFrac distance metrics. The PERMANOVA test on beta diversity (999 permutations) was applied to compare the microbial community dissimilarity across groups [99]. Adnois was applied to investigate the microbial community dissimilarity across age, gender, weight and BMI. The co-occurrence/co-exclusion network was inferred by the Sparse and Compositionally Robust Inference of Microbial Ecological Networks (SPIEC-EASI) framework using generalized LASSO regression (‘glasso’) [100].

### 4.5. Quantitative Real Time PCR

The remaining fecal DNA from 16S rRNA sequencing was retrieved for analysis. Real-Time PCR was carried out with a total volume of 10 μL, containing 5 μL of GoTaq qPCR master mix (Promega Corporation, Waltham, MA USA), 2 μL of DNA template, and 3 μL of primer pair solution (300 nM/reaction). For each run, nuclease-free water (Promega Corporation, Waltham, USA) was used as the negative control, and melting peaks were used to determine the specificity of the PCR. qPCR was performed in a DNA thermal cycler (QuantStudio 1 Real-Time PCR System, Thermo Fisher Scientific, Waltham, USA). PCR conditions included an initial denaturation step at 95 °C for 2 min, followed by 40 cycles consisting of 95 °C for 15 s and 60 °C for 1 min, and an additional dissociation step (95 °C for 15 s, 60 °C for 1 min, followed by a slow ramp to 95 °C). The primer sequences are included in Table S6.

### 4.6. Development of Gut Microbiome Dysbiosis Index (MDI)

All machine learning training was conducted in Python 3.9.13 with scikit learn version 1.1.1, xgboost version 1.7.1, lgbm version 3.3.3, and shap version 0.41.0. The data set was first split into a training and testing set in a 3:1 ratio with stratification. Machine learning models (LR—Logistic Regression, SVM—Support Vector Machine, RF—Random Forest, XGB—Xtreme Gradient Boosting, LGBM—Light Gradient Boosting Machine) were trained using the training set with 4-fold cross-validation to evaluate the F1 score, precision, recall and area under ROC. The model was then tested against the 25% held-out testing set. The best model was selected to be the final model. Feature importance was evaluated by SHAP values and calculated using the shap package.

### 4.7. Statistical Analysis

All the statistical analysis and visualization of results were conducted in Python 3.8.13 with numpy version 1.22.3, scipy version 1.8.0, statsmodels version 0.13.2, skbio version 0.5.6, matplotlib version 3.5.1 and seaborn version 0.11.2. Normality assumptions were evaluated by D’Agostino and Pearson’s test (scipy.stats.normaltest function) and the Shapiro–Wilk test (scipy.stats.shapiro function) if parametric tests were employed. Demographic characteristics were evaluated by the non-parametric Mann–Whitney U rank test (scipy.stats.mannwhitneyu function) for continuous variables and the Fisher exact test for categorical variables (scipy.stats.fisher_exact function). *p*-value correction was performed with statsmodels.stats.multitest.multipletests function using Benjamini/Hochberg (non-negative) procedure. *p* < 0.05 was considered statistically significant unless otherwise specified.

## 5. Conclusions

We demonstrated the notable changes in the gut microbiome composition (1) between psoriasis and control group and (2) psoriatic patients taking 8 weeks of probiotics in a local cohort consisting of southern Chinese patients. *B. wexlerae* and *P. distasonis* were commonly dysregulated species in psoriasis patients. The remodeling of functional pathways and the co-occurrence network was evidential and could provide a novel intuition of the underlying logic and role of gut microbiota in psoriasis development.

## Figures and Tables

**Table 1:** Baseline demographic and disease characteristics of participants.

		Psoriasis (*n* = 58 ^^^)	Control (*n* = 49)	*p* Value
Sex, No. (%)				0.0122
	Male	38 (65.5)	20 (40.8%)	
	Female	20 (34.5)	29 (59.2%)	
Age, mean (SD) (range), y		44.4 (12.1) (18–65)	45.5 (13.2) (21–64)	0.4624
Weight, mean (SD), kg		71.8 (16.3)	63.1 (13.4)	0.0026
BMI, mean (SD) ^†^		25.8 (4.8)	23.4 (4.0)	0.0049
BSA, mean (SD)		13.1 (16.2)	N/A	
PASI, mean (SD)		7.6 (7.1)	N/A	
DLQI, mean (SD)		7.6 (7.1)	N/A	
Psoriatic arthritis, No. (%)		20 (34.5)	N/A	

^†^ BMI between 25.0 and 29.9 kg/m^2^ is classified as overweight, while BMI > 29.9 kg/m^2^ is classified as obese. ^^^ Six participants with psoriasis lost to follow up at week 8. BMI, body mass index; PASI, Psoriasis Area and Severity Index; DLQI, Dermatology Life Quality Index.

**Table 2:** Differentially abundant ASV (taxonomical unit assigned by q2-feature-classifier) identified by ANCOM.

Feature ID	Taxon	W
01f2a9a0f64a0c8e9f380e4759f68e42	f_Bacteroidaceae;g_Bacteroides;s_Bacteroides_vulgatus	4972
ccc00ff50c15bc9d115d53589fd1db6f	f_Lachnospiraceae;g_Fusicatenibacter;s_uncultured_organism	4945
725ae09bd27ee835f1e2afaee85aafe0	f_Lachnospiraceae;g_Blautia;s_Blautia_wexlerae	4940
16feabdbf1087e04e1eef52210ed93ad	f_Erysipelatoclostridiaceae;g_Erysipelotrichaceae_UCG-003;s_uncultured_bacterium	4918
7523ad91056301e66e3a8f299c0878fc	f_Ruminococcaceae;g_Faecalibacterium	4905
f678835c929c59443b1e5e736048e209	f_Ruminococcaceae;g_Faecalibacterium;s_human_gut	4903
fd947e8f706a04316b167e2935696c96	f_Lachnospiraceae;g_Dorea	4896
f86c8767307c0f2ab691b9892c1e99c6	f_Lachnospiraceae;g_Agathobacter;s_[Eubacterium]_rectale	4890
d37e5aa22497be27a11bce9eda0c8cf7	f_Lachnospiraceae;g_Blautia;s_Blautia_wexlerae	4875
da5fdabeacf9b6cafc8d2dcbe81db05d	f_Bacteroidaceae;g_Bacteroides;s_Bacteroides_thetaiotaomicron	4850
18a8fe3a9227e73e6f15d50980cb5c75	f_Lachnospiraceae;g_Anaerostipes;s_human_gut	4826
428f4904e0dfb8dfb40952a57526dbb2	f_Lachnospiraceae;g_Lachnospiraceae_ND3007_group;s_metagenome	4817
f4465d8e3fddd8dd39702387f21ffb4e	f_Bacteroidaceae;g_Bacteroides;s_Bacteroides_vulgatus	4791
f444b6959a6760ca409a51e8107f729f	f_Ruminococcaceae;g_Subdoligranulum	4772
3866fc184b629f22b6b29f848dd90694	f_Lachnospiraceae;g_Blautia	4765
bf5fc5be757c244017374074589e41d7	f_Monoglobaceae;g_Monoglobus;s_uncultured_organism	4745
7c73dc21566ae02ef3e98859d879468c	f_Lachnospiraceae;g_[Ruminococcus]_torques_group;s_uncultured_Firmicutes	4728
a0836bd4a80786ce759ffb26d563eb54	f_Lachnospiraceae;g_Blautia;s_uncultured_Blautia	4686
928a2600b168d35b17d8c7578352c596	f_Streptococcaceae;g_Streptococcus;s_Streptococcus_salivarius	4647
9755d76a1a899b96090b30fe9582c058	f_Butyricicoccaceae;g_Butyricicoccus	4634
60d6ecdf998b630c36e1277da586e204	f_Ruminococcaceae;g_Faecalibacterium	4621
434c8851e06baea123e89217030e6e23	f_Bacteroidaceae;g_Bacteroides;s_Bacteroides_uniformis	4620
93198e1fc1b71b9e7d3abf522a926278	f_Lachnospiraceae;g_Blautia;s_Blautia_faecis	4588
65b9f8997ba78cf0dae03e7146efc23b	f_Lachnospiraceae;g_Blautia;s_Blautia_wexlerae	4543
4655a784c774d4acb1bb902a65ed9612	f_Lachnospiraceae;g_Lachnoclostridium	4483

**Table 3:** Demographic and disease characteristics of psoriatic participants.

		Mild ^&^ (*n* = 20)	Moderate to Severe ^&^ (*n* = 32)	*p* Value
Sex, No. (%)				0.2237
	Male	11 (55.0)	24 (75.0)	
	Female	9 (45.0)	8 (25.0)	
Age, mean (SD), y		44.1 (13.5)	44.2 (10.8)	0.9795
Weight, mean (SD), kg		68.9 (16.6)	75.3 (15.8)	0.1722
BMI, mean (SD) ^†^		25.4 (5.8)	26.5 (4.4)	0.4200
Psoriatic arthritis, No. (%)		6 (30.0)	10 (31.3)	1.0000
∆BSA, mean (SD)		−2.1 (2.1)	−5.8 (12.1)	0.1925
∆%PASI, mean (SD)		−21.7 (15.9)	−26.5 (31.4)	0.7692
∆DLQI, mean (SD)		−2.6 (6.5)	−2.1 (6.2)	0.5443
Responder, No. (%) ^^^		8 (40.0)	14 (43.8)	1.0000

^†^ BMI between 25.0 and 29.9 kg/m^2^ is classified as overweight, while BMI > 29.9 kg/m^2^ is classified as obese. ^&^ Those who were either on biologics or PASI > 10 at the baseline were regarded as having moderate to severe psoriasis. ^^^ Those who showed significant improvement in PASI or DLQI were regarded as responders. BMI, body mass index; PASI, Psoriasis Area and Severity Index; DLQI, Dermatology Life Quality Index.

**Table 4:** Differentially abundant ASV (taxonomical unit assigned by q2-feature-classifier) identified by ANCOM after the course of 8-week oral administered probiotics.

Feature_ID	Taxon	W
721a741bef4a5db0211de1a5b84a8b5b	f_Tannerellaceae;g_Parabacteroides;s_Parabacteroides_distasonis	4139
72af122f180225b2b0c90e487b25af6c	f_Lactobacillaceae;g_Lactobacillus;s_Lactobacillus_plantarum	4139
8cec479da287209d3c7d464f14242794	f_Ruminococcaceae;g_CAG-352;s_uncultured_bacterium	4139
bd4606ad663e209e745c8c51b4deeee8	f_Bacteroidaceae;g_Bacteroides;s_Bacteroides_vulgatus	4142
f678835c929c59443b1e5e736048e209	f_Ruminococcaceae;g_Faecalibacterium;s_human_gut	4141

**Table 5:** Differentially abundant MetaCyc pathways and its corresponding LDA score and *p* value inferred by PICRUSt2 and LefSe.

BioCyc ID	MetaCyc Pathway Name	Group	Log LDA	*p*
PWY_5989	stearate biosynthesis II (bacteria and plants)	control	2.7092	0.0128
PWYG_321	mycolate biosynthesis	control	2.6975	0.0113
PWY_7664	oleate biosynthesis IV (anaerobic)	control	2.6970	0.0140
PWY_6282	palmitoleate biosynthesis I (from (5Z)-dodec-5-enoate)	control	2.6923	0.0138
PWY0_862	(5Z)-dodecenoate biosynthesis I	control	2.6826	0.0142
PWY_6519	8-amino-7-oxononanoate biosynthesis I	control	2.6193	0.0037
FASYN_ INITIAL_PWY	super pathway of fatty acid biosynthesis initiation	control	2.5984	0.0140
P42_PWY	incomplete reductive TCA cycle	control	2.5914	0.0024
BIOTIN_ BIOSYNTHESIS_PWY	biotin biosynthesis I	control	2.5560	0.0096
PWY_6703	preQ_0_ biosynthesis	control	2.3734	0.0252
THISYN_PWY	super pathway of thiamine diphosphate biosynthesis I	control	2.3712	0.0059
HISDEG_PWY	l-histidine degradation I	control	2.3484	0.0070
PWY_7323	super pathway of GDP-mannose-derived o-antigen building blocks biosynthesis	control	2.3441	0.0074
PWY_5695	inosine 5′-phosphate degradation	control	2.3388	0.0282
NAGLIPASYN_PWY	lipid IV_A_ biosynthesis (*E. coli*)	control	2.3144	0.0244
GALACTUROCAT_PWY	d-galacturonate degradation I	control	2.3052	0.0175
POLYISOPRENSYN_PWY	polyisoprenoid biosynthesis (*E. coli*)	control	2.2352	0.0305
PWY_6467	Kdo transfer to lipid IV_A_ (*Chlamydia*)	control	2.1935	0.0228
PWY_7328	super pathway of UDP-glucose-derived o-antigen building blocks biosynthesis	control	2.1563	0.0184
PWY_4984	urea cycle	control	2.1505	0.0204
COLANSYN_PWY	colanic acid building blocks biosynthesis	control	2.1109	0.0166
ASPASN_PWY	super pathway of l-aspartate and l-asparagine biosynthesis	control	2.0583	0.0305
P161_PWY	acetylene degradation (anaerobic)	psoriasis	2.5697	0.0074
PWY0_1586	peptidoglycan maturation (*meso*-diaminopimelate containing)	psoriasis	2.5201	0.0300
PWY_6471	peptidoglycan biosynthesis IV (*Enterococcus faecium*)	psoriasis	2.4510	0.0184
PWY_5505	l-glutamate and l-glutamine biosynthesis	psoriasis	2.3867	0.0330
NONOXIPENT_PWY	pentose phosphate pathway (non-oxidative branch) I	psoriasis	2.3355	0.0454
PWY_7111	pyruvate fermentation to isobutanol (engineered)	psoriasis	2.3156	0.0296
PWY0_1297	super pathway of purine deoxyribonucleosides degradation	psoriasis	2.3149	0.0126
PWY_6151	*S*-adenosyl-l-methionine salvage I	psoriasis	2.3130	0.0210
PWY_621	sucrose degradation III (sucrose invertase)	psoriasis	2.3016	0.0207
PWY_5686	UMP biosynthesis I	psoriasis	2.2906	0.0296
PWY_6317	d-galactose degradation I (Leloir pathway)	psoriasis	2.2897	0.0128
DAPLYSINESYN_PWY	l-lysine biosynthesis I	psoriasis	2.2642	0.0320
PWY0_1298	super pathway of pyrimidine deoxyribonucleosides degradation	psoriasis	2.2569	0.0138
ARGSYNBSUB_PWY	l-arginine biosynthesis II (acetyl cycle)	psoriasis	2.2303	0.0286
ARGSYN_PWY	l-arginine biosynthesis I (via L-ornithine)	psoriasis	2.2162	0.0277
PWY_7400	l-arginine biosynthesis IV (archaea)	psoriasis	2.2090	0.0277
COMPLETE_ARO_PWY	super pathway of aromatic amino acid biosynthesis	psoriasis	2.1875	0.0391
ARO_PWY	chorismate biosynthesis I	psoriasis	2.1698	0.0482
SER_GLYSYN_PWY	super pathway of l-serine and glycine biosynthesis I	psoriasis	2.1284	0.0273
PWY_6163	chorismate biosynthesis from 3-dehydroquinate	psoriasis	2.1129	0.0403
PWY_6122	5-aminoimidazole ribonucleotide biosynthesis II	psoriasis	2.0896	0.0373
PWY_6277	super pathway of 5-aminoimidazole ribonucleotide biosynthesis	psoriasis	2.0896	0.0373

## Data Availability

The raw sequence data are available in NCBI (PRJNA934420). Due to the restriction of consent and sensitivity, the metadata and qPCR data are available upon reasonable request made to the corresponding authors.

## Supplementary Materials

The supporting information can be downloaded at: https://www.mdpi.com/article/10.3390/ijms24076571/s1.
