# Supplementary material for: A Novel E3 Probiotics Formula Restored Gut Dysbiosis and Remodelled Gut Microbial Network and Microbiome Dysbiosis Index (MDI) in Southern Chinese Adult Psoriasis Patients
Source: Int J Mol Sci. 2023 Mar 31;24(7):6571. doi: 10.3390/ijms24076571 (PMC10094986; doi:10.3390/ijms24076571)
Supplement: Supplementary file 1 [file ijms-24-06571-s001.zip › ijms-2287884-supplementary/psoriasis-ijms-suppl-proofread/Figures and Tables caption.pdf]

Supplementary Figure S1. Alpha–rarefaction curve based on (A) observed features and (B) Shannon's index.
Supplementary Figure S2. Boxplot of alpha diversity metrics between control and psoriasis group (Mann–Whitney U test): (A) ACE, (B) Chao1, (C) Faith's phylogenetic diversity (Faith PD), (D) observed features, (E) Shannon's index, and (F) inverse Simpson.
Supplementary Figure S3. Boxplot of alpha diversity metrics of psoriatic participants (Wilcoxon signed rank): (A) ACE, (B) Chao1, (C) Faith's phylogenetic diversity (Faith PD), (D) observed features, (E) Shannon's index, and (F) inverse Simpson.
Supplementary Figure S4. Gut composition profile of psoriasis group. (A) Principal Coordinate Analysis biplot based on Bray–Curtis, Jaccard distances, unweighted UniFrac and weighted UniFrac (PERMANOVA, 999 permutations). (B) Relative abundance of phyla. (C) Venn diagram of all ASV. (D) Venn diagram of ASV (excluding rare ASV).
Supplementary Figure S5. Boxplot of clr transformed abundance of (A) phylum Synergistota. (B) Lactobacillus acidophilus. (C) Blautia wexlerae.
Supplementary Figure S6. Boxplot of clr transformed abundance of the ASV corresponding to *P. distasonis*.
Supplementary Figure S7. Boxplot of clr transformed abundance of COLANSYN-PWY.
Supplementary Figure S8. Training performance of machine learning model of (A) 10 features. (B) top 5 most important features (reduced dimensionality).
Supplementary Figure S9. Feature importance of RF, XGB and LGBM.
Supplementary Figure S10. Distribution of the microbial markers by (A) clr transformed abundance. (B) log 2 ΔCt.
Supplementary Table S1. Beta diversity stratified by demographic characteristics regardless of grouping.
Supplementary Table S2. Confidence of taxonomic assignment of *Parabacteroides distasonis*.
Supplementary Table S3. Common edges shared by control and psoriasis network.
Supplementary Table S4. Common edges shared by psoriasis network at baseline and at week 8.
Supplementary Table S5. Common edges shared by control and psoriasis network at week 8.
Supplementary Table S6. qPCR primer sequences of the microbial markers.
Supplementary Table S7. Improvement in PASI or DLQI of psoriatic participants.
