# Supplementary material for: A Novel E3 Probiotics Formula Restored Gut Dysbiosis and Remodelled Gut Microbial Network and Microbiome Dysbiosis Index (MDI) in Southern Chinese Adult Psoriasis Patients
Source: Int J Mol Sci. 2023 Mar 31;24(7):6571. doi: 10.3390/ijms24076571 (PMC10094986; doi:10.3390/ijms24076571)

Supplementary Figure S1

A

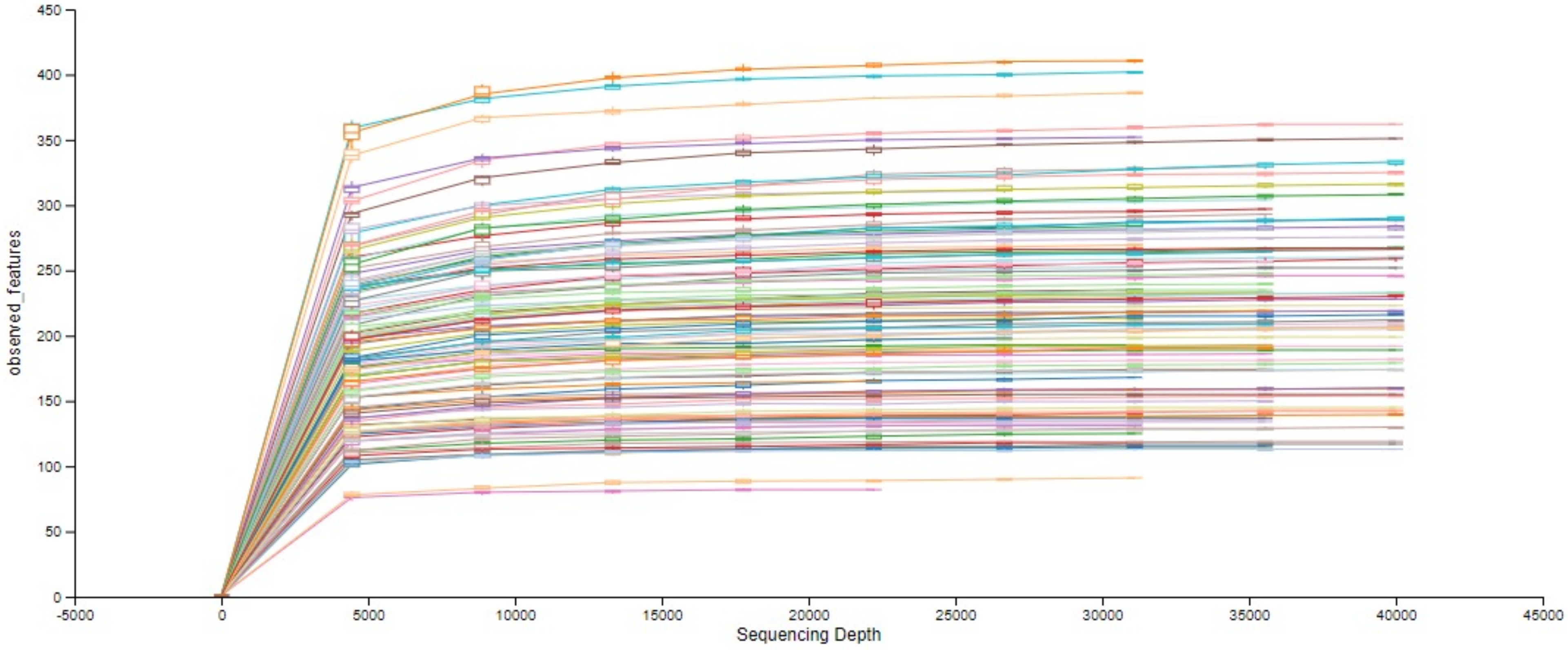

B

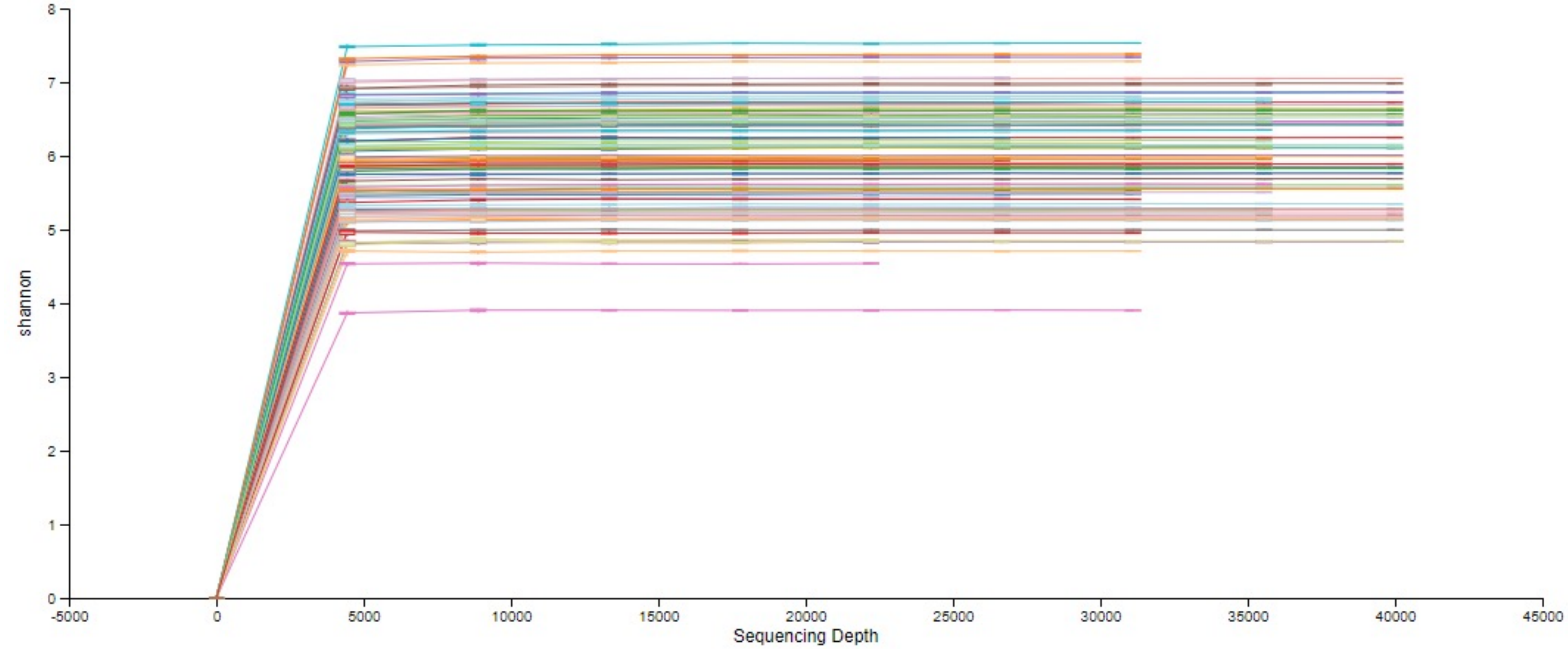

Supplementary Figure S2

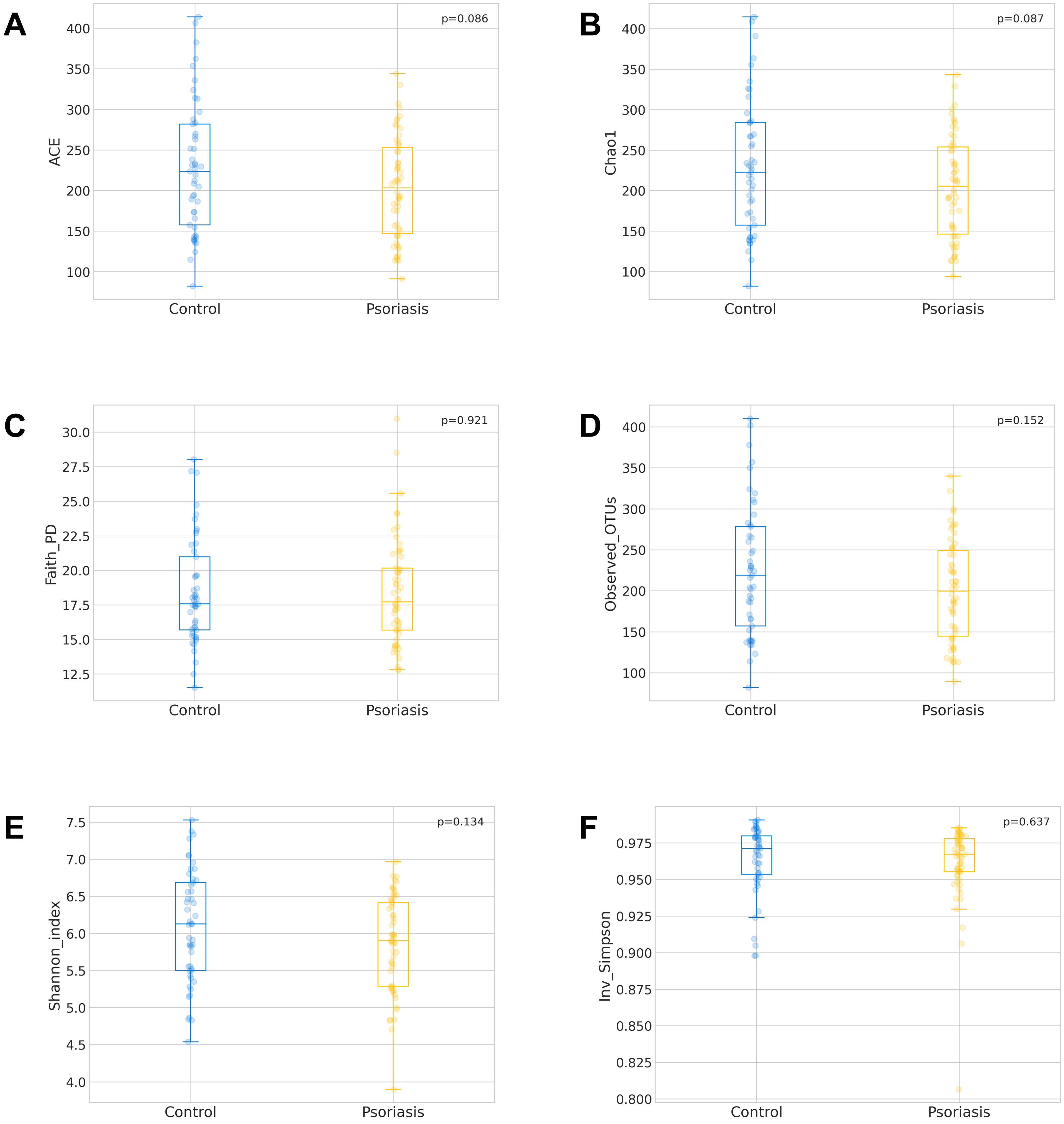

Supplementary Figure S3

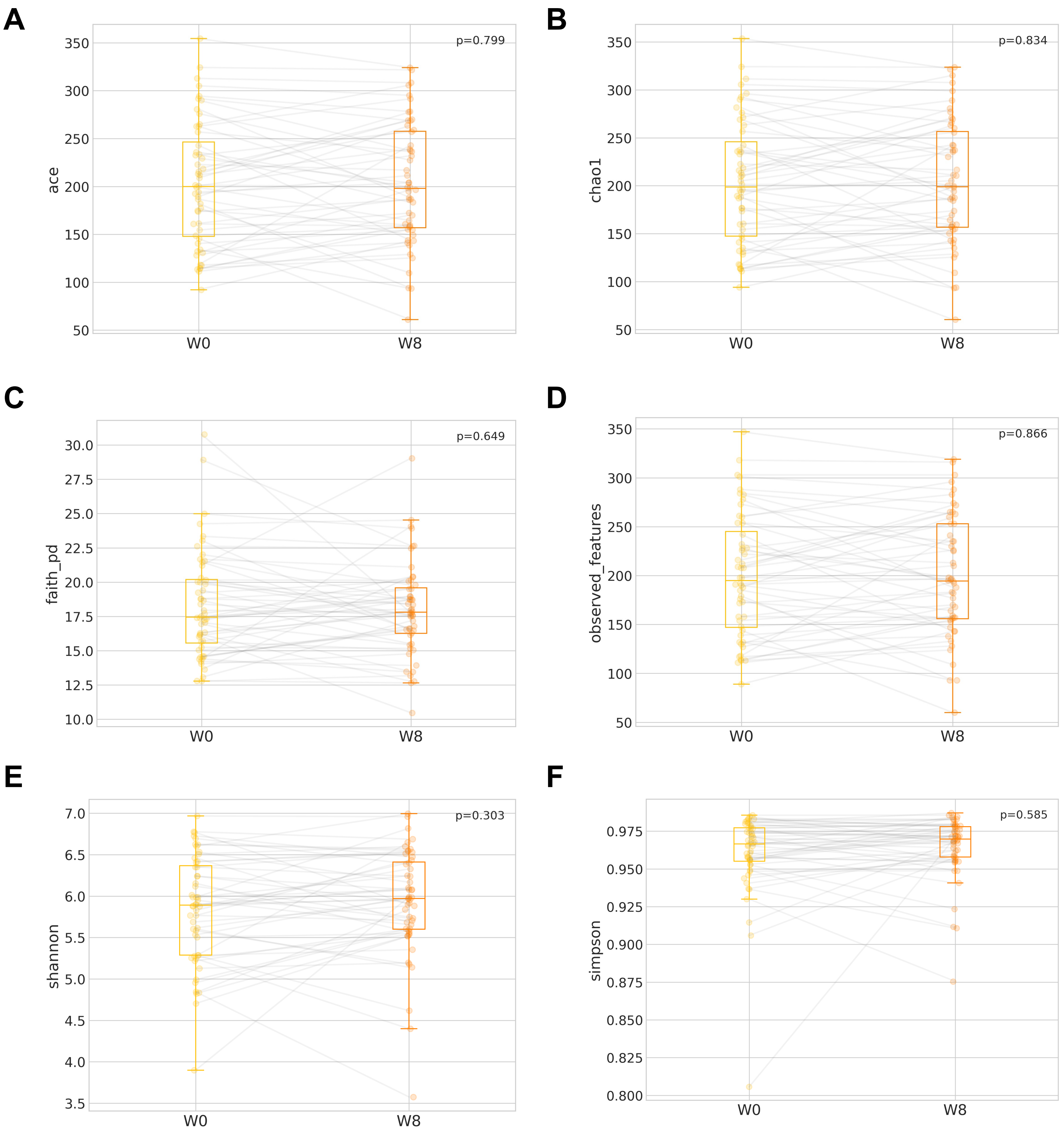

Supplementary Figure S4

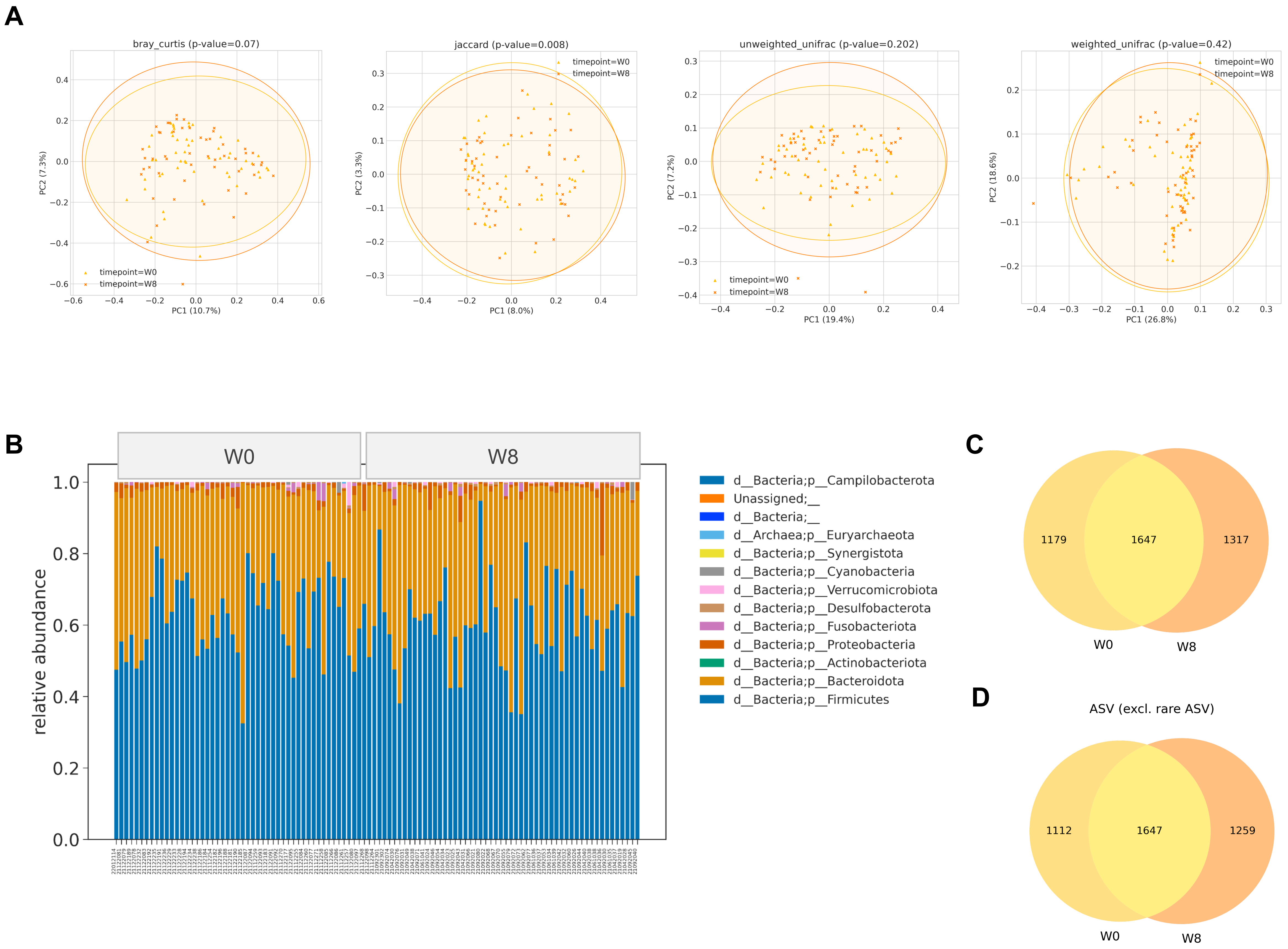

Supplementary Figure S5

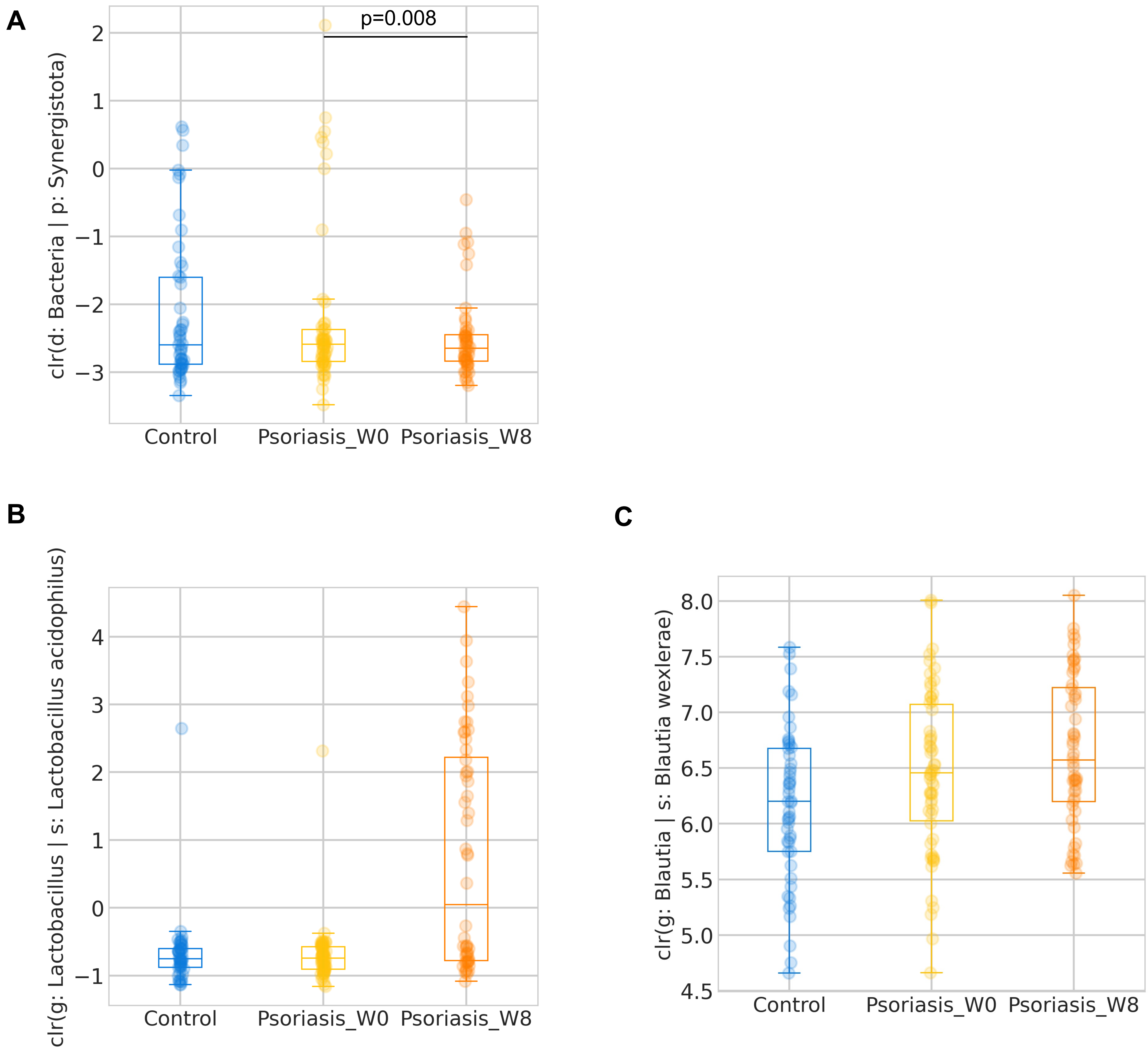

Supplementary Figure S6

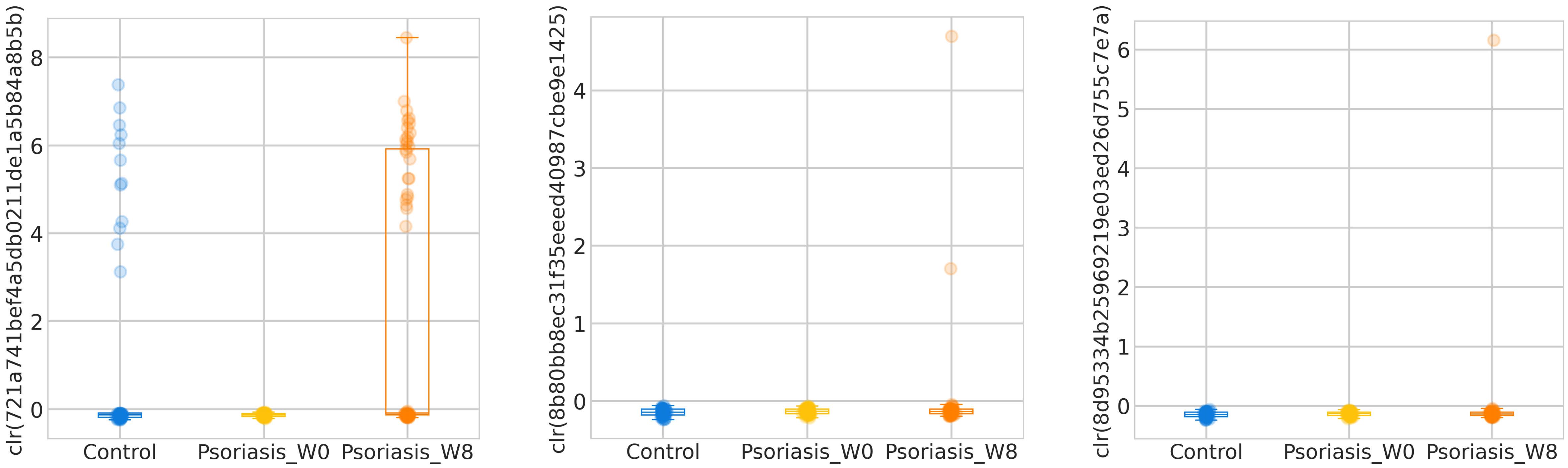

Supplementary Figure S7

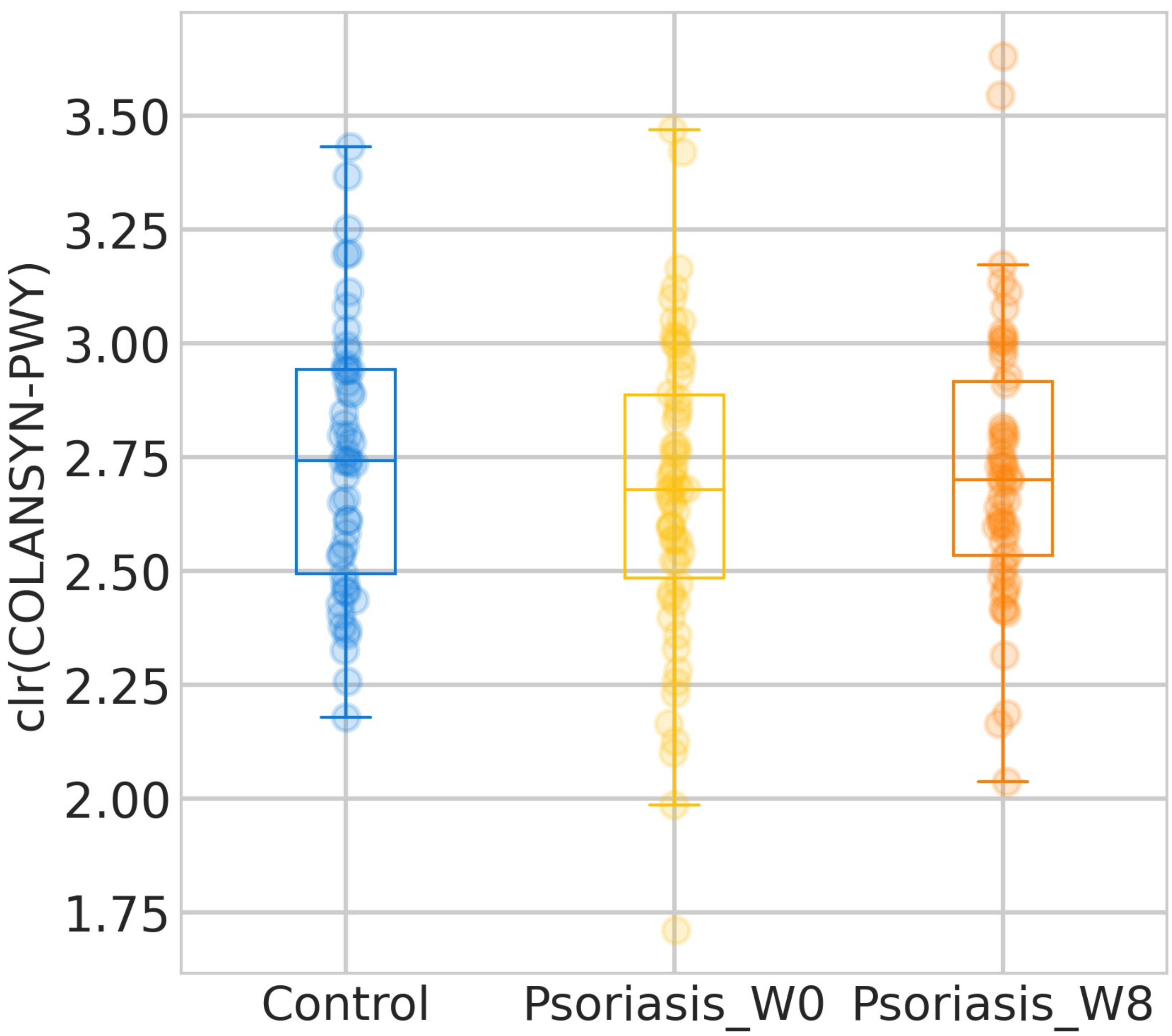

Supplementary Figure S8

A

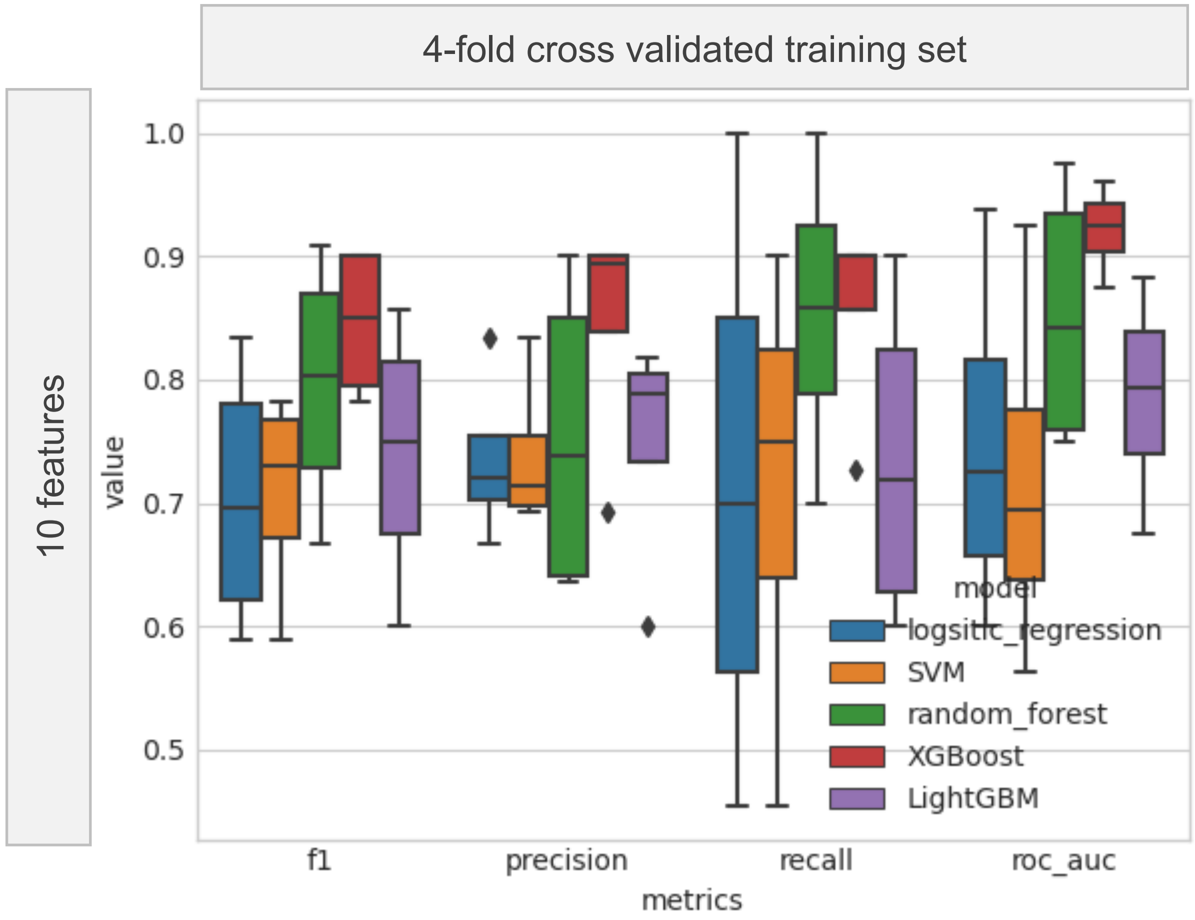

25% held out test set

	F	Precision	Recall	AUROC
**LR**	0.78	1.00	0.64	0.82
**SVM**	0.83	1.00	0.71	0.86
**RF**	0.89	0.92	0.86	0.88
**XGB**	0.86	0.86	0.86	0.83
**LGBM**	0.89	0.92	0.86	0.88

B

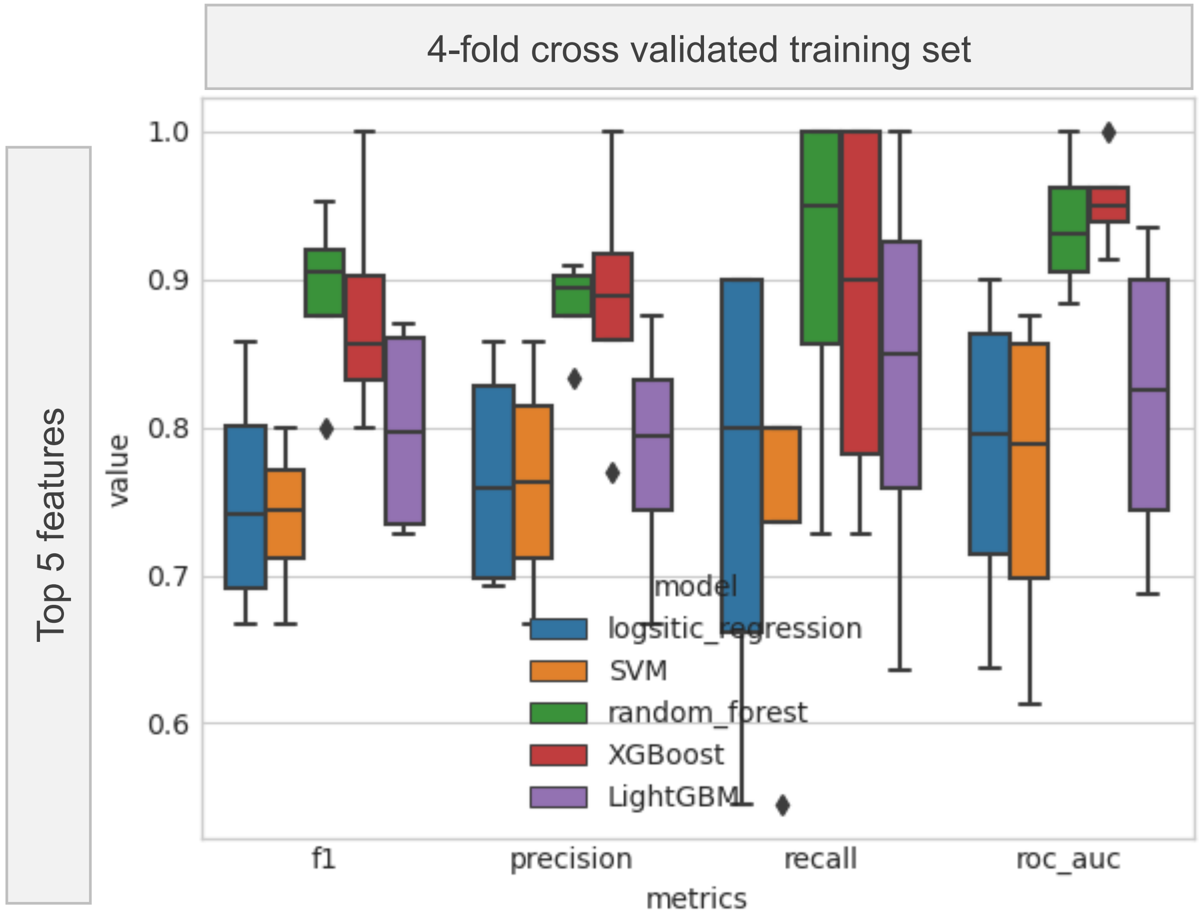

25% held out test set

	F	Precision	Recall	AUROC
**LR**	0.78	1.00	0.64	0.82
**SVM**	0.78	1.00	0.64	0.82
**RF**	0.87	0.82	0.93	0.81
**XGB**	0.86	0.86	0.86	0.83
**LGBM**	0.89	0.92	0.86	0.88

Supplementary Figure S9

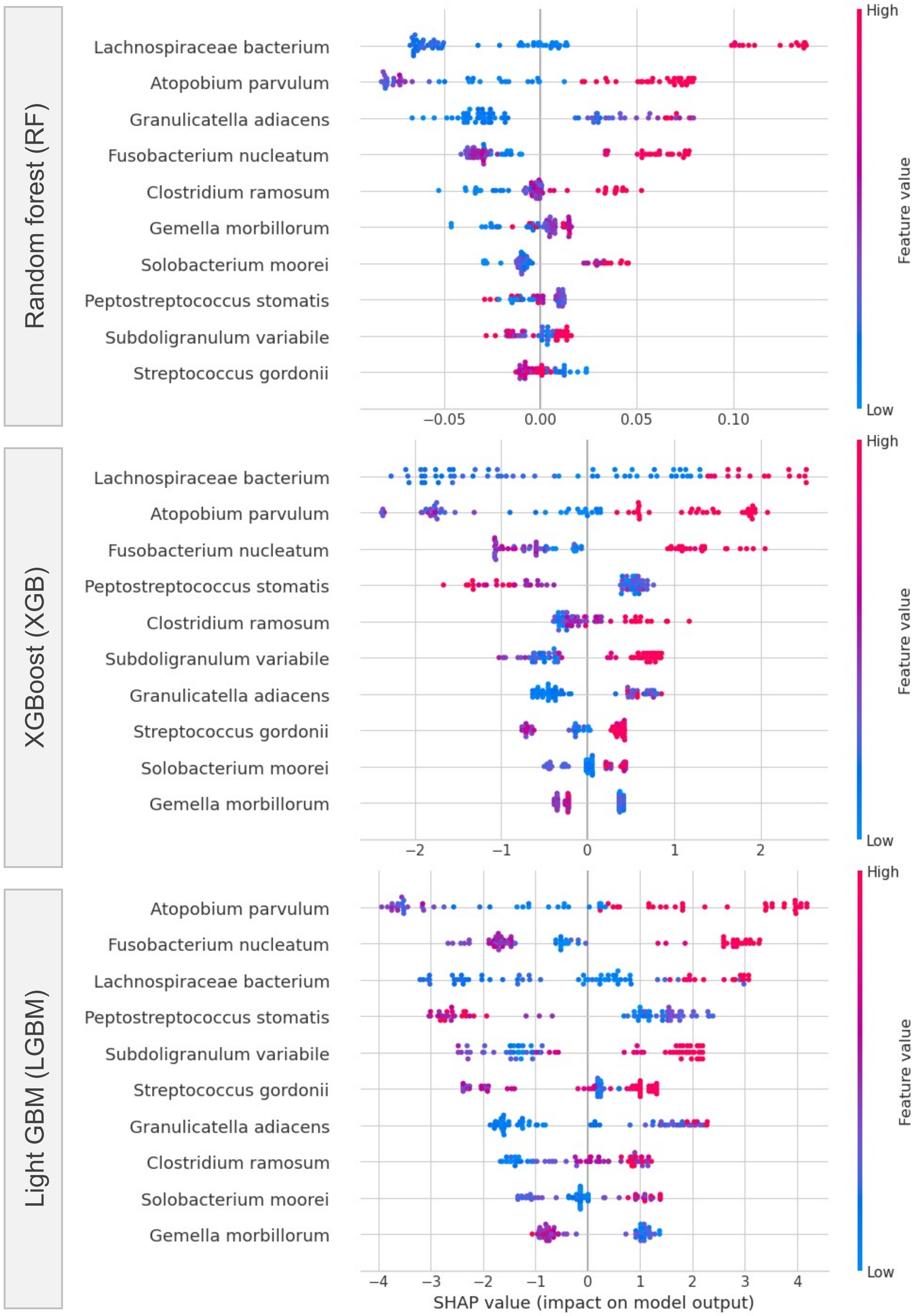

Supplementary Figure S10

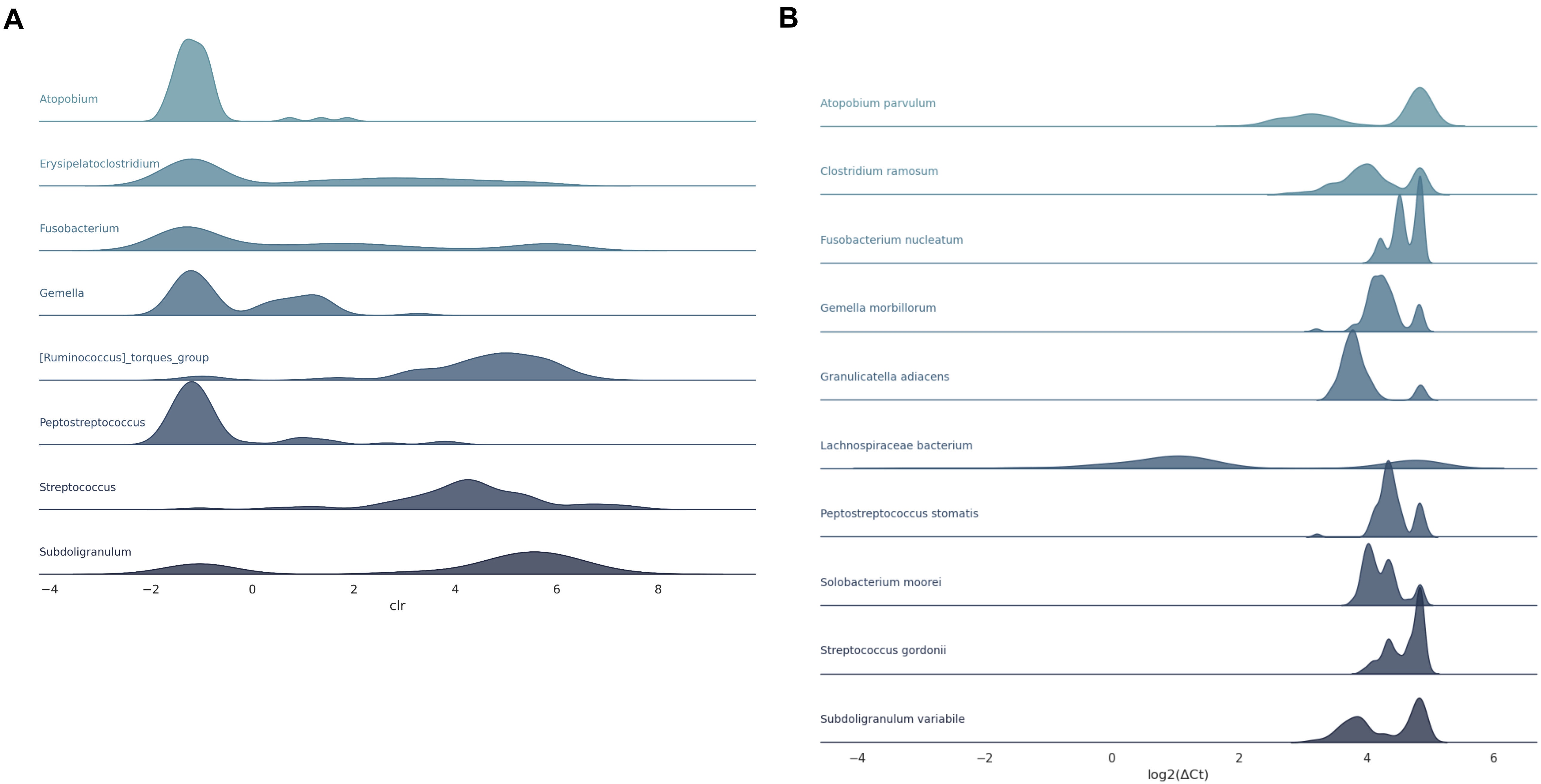

Supplement: Supplementary file 1 [file ijms-24-06571-s001.zip › ijms-2287884-supplementary/psoriasis-ijms-suppl-proofread/psoriasis_suppl-figures_20230331.pdf]
